# The protective effect of low-dose minocycline on brain microvascular ultrastructure in a rodent model of subarachnoid hemorrhage

**DOI:** 10.1007/s00418-022-02150-9

**Published:** 2022-09-24

**Authors:** Daria Gendosz de Carrillo, Sebastian Student, Daniel Bula, Łukasz Mielańczyk, Małgorzata Burek, Patrick Meybohm, Halina Jędrzejowska-Szypułka

**Affiliations:** 1 Department of Physiology, Faculty of Medical Sciences in Katowice, Medical University of Silesia, Poniatowskiego 15, 40-055 Katowice, Poland; 2 Department of Histology and Cell Pathology, Faculty of Medical Sciences in Zabrze, Medical University of Silesia, Poniatowskiego 15, 40-055 Katowice, Poland; 3 Department of Systems Biology and Engineering, Silesian University of Technology, Gliwice, Poland; 4 Biotechnology Centre, Silesian University of Technology, Gliwice, Poland; 5 Oncological and Reconstructive Surgery Department, Gliwice Branch, Maria Sklodowska-Curie National Research Institute of Oncology, Gliwice, Poland; 6 Department of Anaesthesiology, Intensive Care, Emergency and Pain Medicine, University Hospital Würzburg, Würzburg, Germany

**Keywords:** Subarachnoid hemorrhage, Minocycline, Ultrastructure, Blood–brain barrier

## Abstract

The multifaceted nature of subarachnoid hemorrhage (SAH) pathogenesis is poorly understood. To date, no pharmacological agent has been found to be efficacious for the prevention of brain injury when used for acute SAH intervention. This study was undertaken to evaluate the beneficial effects of low-dose neuroprotective agent minocycline on brain microvascular ultrastructures that have not been studied in detail. We studied SAH brain injury using an in vivo prechiasmatic subarachnoid hemorrhage rodent model. We analyzed the qualitative and quantitative ultrastructural morphology of capillaries and surrounding neuropil in the rodent brains with SAH and/or minocycline administration. Here, we report that low-dose minocycline (1 mg/kg) displayed protective effects on capillaries and surrounding cells from significant SAH-induced changes. Ultrastructural morphology analysis revealed also that minocycline stopped endothelial cells from abnormal production of vacuoles and vesicles that compromise blood–brain barrier (BBB) transcellular transport. The reported ultrastructural abnormalities as well as neuroprotective effects of minocycline during SAH were not directly mediated by inhibition of MMP-2, MMP-9, or EMMPRIN. However, SAH brain tissue treated with minocycline was protected from development of other morphological features associated with oxidative stress and the presence of immune cells in the perivascular space. These data advance the knowledge on the effect of SAH on brain tissue ultrastructure in an SAH rodent model and the neuroprotective effect of minocycline when administered in low doses.

**Supplementary Information:**

The online version contains supplementary material available at 10.1007/s00418-022-02150-9.

## Introduction

Subarachnoid hemorrhage (SAH) is a devastating cerebrovascular disease associated with a complex, multisystem, and multifaceted pathogenic mechanism involving several processes, which are sometimes independent actions, resulting in cell death (Chen et al. 2014). SAH accounts for 5% of all stroke incidents, and 85% of SAH incidents are caused by spontaneous, nontraumatic aneurysm ruptures, primarily located at the bifurcation of Willis polygon vessels and their branches (Chen et al. 2014). The blood released from a ruptured aneurysm into the subarachnoid space surrounding the brain can affect blood flow and, together with toxic hemoglobin breakdown products, can significantly disrupt the conditions of the vascular environment, wreaking havoc associated with brain tissue damage during early brain injury (Tso and Macdonald 2014; Macdonald and Schweizer 2017). Despite the high morbidity and disability rate associated with SAH, no pharmacologic agent has been shown to be effective for the prevention of early brain injury during acute SAH interventions (Chen et al. 2014; Suzuki and Nakano 2018).

The sustained and monitored transport of nutrients, via paracellular and transcellular pathways, is crucial for the maintenance of brain homeostasis. Therefore, the loss of BBB integrity is associated with altered BBB permeability (Salvador et al. 2021). Particular attention has been focused on the roles played by tight junctions (TJs) and the proteins that constitute the TJ complex during SAH pathogenesis (Peeyush Kumar et al. 2019; Kanamaru and Suzuki 2019). However, electron microscopy evidence demonstrating the impacts of TJ disassembly impact on BBB function in the context of ischemia has been inconsistent (Krueger et al. 2013; Knowland et al. 2014; Nahirney et al. 2016), and the contribution of disarrayed transport through the endothelium following brain tissue damage has recently become more apparent (Nahirney et al. 2016); however, this process has not been examined in SAH. In addition to BBB constituents, hemorrhage strikes the remaining components of the neurovascular unit (NVU) and profoundly impacts communications between neurons, astrocytes, and arterioles, causing the inversion of neurovascular coupling; therefore, when demand for oxygen and glucose in neurons increases, capillaries constrict instead of dilating (Balbi et al. 2017).

Matrix metalloproteinases (MMPs) play a significant role in the homeostasis of brain tissue and capillaries. Since their discovery, MMP-2 and MMP-9 have been extensively studied both in brain physiology and pathology and became an important target for neuroprotective therapies in acute brain diseases. The role for neuroprotective agents is to improve clinical conditions and alleviate neurological deficits in patients affected by brain injuries by protecting brain cells from the pathological mechanisms that can cause damage as early as ictus onset. Tetracycline and its derivatives, such as minocycline and doxycycline, are semisynthetic antibiotics that have been used as anti-infective agents. In addition to their antimicrobial efficacy, they have been shown to be efficient inhibitors of MMPs, owing to their ability to chelate their cofactors, metal ions. Doxycycline exerts stronger inhibitory effect on MMPs; however, minocycline displays excellent penetration of tissues, owing to its high lipophilicity. Minocycline readily crosses the BBB, and accumulates in cells and in the extracellular matrix of the central nervous system (CNS) (Garrido-Mesa et al. 2013). The neuroprotective efficacy of minocycline has been demonstrated in a variety of animal models of acute neurological injury (Kim and Suh 2009; Garrido-Mesa et al. 2013). In addition, the beneficial effects of minocycline on functional outcomes following SAH have been found to be mediated by MMP-9 activity against SAH-induced vasospasms (Vellimana et al. 2017) and neurobehavioral deficits, in long-term studies using an endovascular model of SAH (Sherchan et al. 2011; Vellimana et al. 2017). Extracellular matrix metalloproteinase inducer (EMMPRIN), an upstream regulator of MMP-2 and MMP-9, has also been reported to be associated with acute microvascular changes after SAH (Hang et al. 2012).

The essential and unresolved knowledge gap in the existing literature concerns the effects of minocycline on the morphology of brain tissues during the acute phase after SAH. To address this problem, we applied a two-step analysis method to characterize the potential of using low-dose minocycline as a neuroprotective treatment for SAH. First, we described the SAH-related qualitative and quantitative changes to the brain ultrastructure. Special attention was paid to the impact of SAH on microcapillaries and surrounding neural tissue. Morphological characteristics of individual cells from NVU were included in our analysis. Second, we defined the parameters that were sensitive to minocycline. In our study, we showed that minocycline protected microcapillaries and cells within perivascular space from morphological alternations related to SAH induction. Minocycline prevented microcapillaries from collapse, and neuropil surrounding capillaries from far-reaching damage. Minocycline also had a protective effect on pericyte and astrocyte morphology, and diminished abnormal vesicle and vacuole production. Together, this analysis provided us with a comprehensive picture of the rat condition 24 h after ictus. Despite the fact that delayed neurological deficits after SAH are uncommon in some models of SAH in rats (Oka et al. 2020), we chose the time of 24 h after ictus because it corresponds to the crucial time between early and delayed injury in humans. We also reported here that SAH injury did not affect the ultrastructure of tight junction (Nahirney et al. 2016), whose morphology was sustained by upregulated expression of *occludin* and *claudin-5* genes. Additionally, we reported that the biochemical changes observed for MMP-2, MMP-9, and their upstream inducer, EMMPRIN, did not contribute to the neuroprotective effects of low-dose minocycline when treating prechiasmatic SAH. However, SAH brain tissue treated with minocycline was protected from cristae degradation within the mitochondria of neuronal cells, and the presence of CD45-positive immune cells in the perivascular space.

In previous studies examining the SAH animal model, the neuroprotective effects of minocycline were obtained when minocycline was administered to experimental animals (Sherchan et al. 2011; Vellimana et al. 2017; Care et al. 2022) at doses exceeding what would be considered the safe application of minocycline to humans (Fagan et al. 2010; Kohler et al. 2013). Therefore, to reliably reflect clinical conditions and reduce the possibility of adverse side effects associated with minocycline administration to patients with SAH, we decided to test the dose of 1 mg/kg/24 h, which corresponds to the clinically tested single dose of 1–1.5 mg/kg (Kohler et al. 2013).

## Methods

### Data availability

All data supporting the findings of this study are available within the paper and Data Supplement. Additional inquires can be directed to the corresponding author.

### Animals

Eighty-six male Wistar rats (350–400 g) were purchased from the breeding facility at the Medical University of Silesia in Katowice, Poland. The rats were maintained in temperature- and humidity-controlled animal quarters, under standard light/dark conditions (12 h/12 h), with food and water ad libitum. The Local Ethical Committee on the Care and Use of Animals for the Medical University of Silesia in Katowice, Poland approved all procedures performed during the study. All animal procedures were performed at Department of Experimental Medicine of Medical University of Silesia in Katowice, Poland.

### Prechiasmatic cistern SAH model

**Fig. 1:**
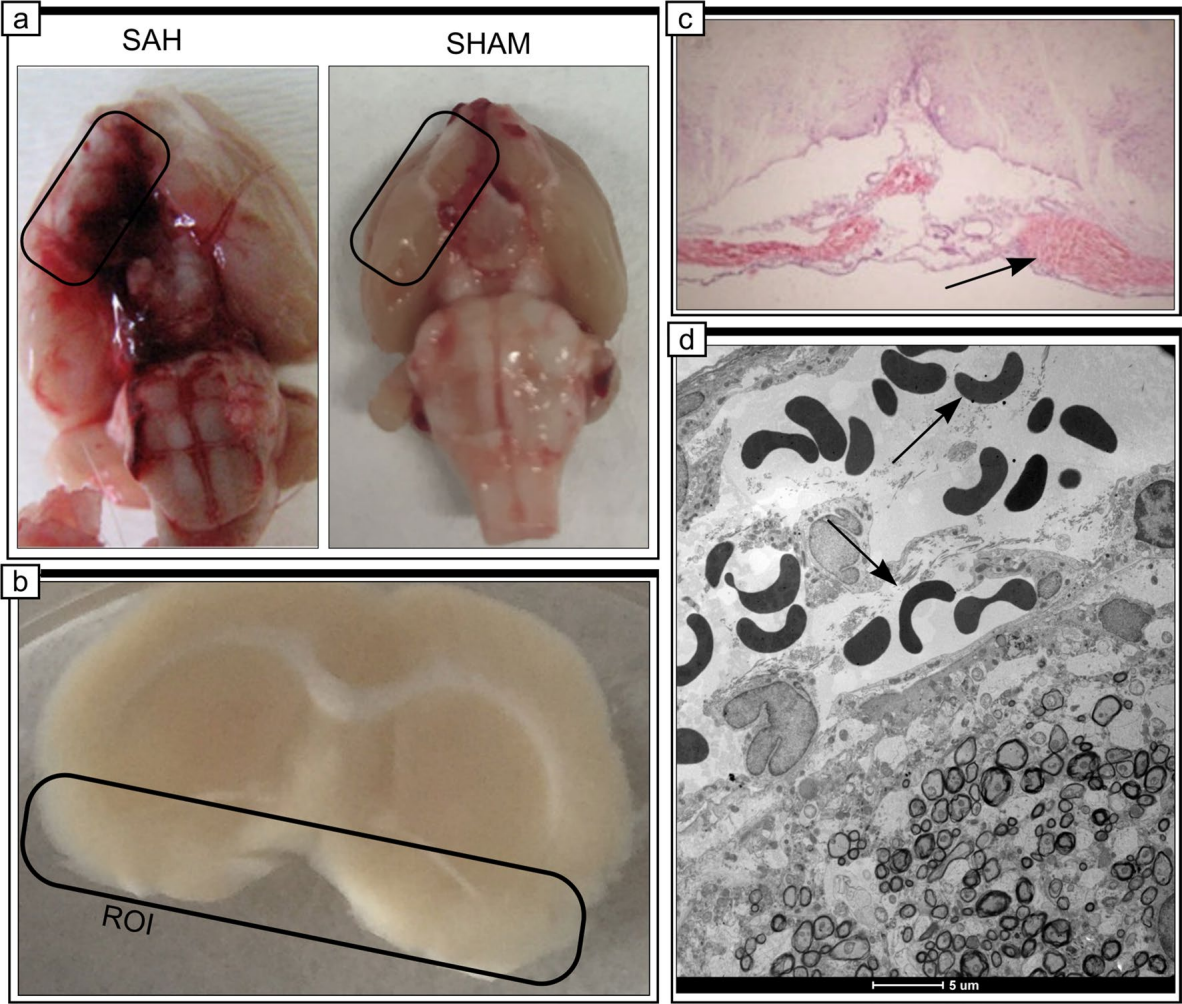
SAH model in rats. **a**, **b** A schematic representation of the areas taken for assay, SAH or SAH + Mino and control rats. **c** Hematoxylin and eosin staining of SAH brain. Black arrow points to hemorrhagic blood present within subarachnoid space of brain. **d** Electron micrograph represents the subarachnoid space filled with erythrocytes (black arrows). Scale bar, 5 μm (**d**)

Eighty-six rats were included in the study. Before surgery, rats received an intraperitoneal injection of a mixture of ketamine (60 mg/kg) and xylazine (10 mg/kg). Then, the rats were placed in a stereotaxic frame, in a prone position. A small incision was made on the top of the skull to expose the midline and bregma. Using a stereomicroscope, a small hole (2 mm diameter) was made, using a dental drill, 0 mm from the midline and 5 mm anterior from bregma. A 0.6-mm-diameter, sterile, heparinized, polyvinyl cannula was inserted into the prechiasmatic cistern, as previously described (Prunell et al. 2002). The tube was tilted 45° in the sagittal plane, placed in the hole, and lowered until the tip of the tube reached the base of the skull, 2 mm anterior to the chiasm. The free outflow of cerebrospinal fluid through the installed catheter verified the correct position of the cannula. Before placing the animal into the supine position, bone wax was applied around the hole in the skull, to avoid the uncontrolled movement of the cannula. Arterial blood (300 µL) was collected from a femoral artery of an animal subjected to the procedure. Directly after blood withdrawal, 250 µL of nonheparinized arterial blood was slowly injected into the prechiasmatic cistern over a 20-s period, under aseptic technique. Then, the cannula was removed, the hole was filled with bone wax, and the skin of the skull was sutured with a surgical thread. Control animals (SHAM) received an injection of 250 µL saline into the prechiasmatic cistern, instead of blood. The animals were subcutaneously injected with 10 mL 0.9% NaCl following the operation, to prevent dehydration, and then returned to their cages. Mortality rate after SAH was 14%. After 24 h from ictus, rats were sacrificed under deep anesthesia and brains were removed. High-resolution pictures of the base of the brains depicting the circle of Willis and basilar arteries were taken to evaluate the extent of SAH using a grading system as previously described (Sugawara et al. 2008; Egashira et al. 2015). The basal brain including brainstem was divided into six segments. Each segment was assigned a grade from 0 to 3, depending on the amount of blood. The minimum SAH grade is 0, and maximum grade is 18. In the present study, only rats that were graded 8 or more were included in the study. We did not observe statistically significant difference between rats from SAH and SAH + minocycline (Mino) groups (data not published). The hemorrhagic blood covered the inferior basal temporal lobe, and brain tissue adjacent to clotted blood was used for later morphological and biochemical analyses (Wang et al. 2012) (Fig. 1).

### Minocycline administration

Rats that underwent SAH induction were randomly divided into SAH or SAH treatment group. Rats from the treatment group received minocycline (Sigma-Aldrich, USA), at a dose of 1 mg/kg, in 1 mL 0.9% NaCl, injected intraperitoneally, 10 min after SAH induction. The animals from the vehicle group, SHAM, received intraperitoneally the same volume of saline 10 min after SAH induction.

### Pain management

Paracetamol in oral suspension was used as a pain relief agent for rats included in the project. One milliliter of paracetamol (100 mg/ml) was added to the 250 ml of drinking water, which was provided ad libitum.

### Transmission electron microscopy

For transmission electron microscopy (TEM) studies, animals (*n* = 3–4 per group) were transcardially perfused, under constant pressure 130 mmHg for 10 min, with 100 mL ice-cold phosphate-buffered saline (PBS), pH 7.4, followed by 200 mL 4% paraformaldehyde solution, pH 7.4. Immediately after brain removal, brain was sliced into 1.5-mm-thick slices and post-fixed in 2.5% glutaraldehyde (SERVA Electrophoresis, Germany) in cacodylate buffer, pH 7.4, for 4 h, at room temperature and then washed several times in the same buffer. Then, tissue samples from cortex within the basal temporal lobe and adjacent to clotted blood from each hemisphere were cut into smaller 1–1.5 mm^3^ blocks (8–12 blocks per animal). Subsequently, the tissue was fixed in 1% osmium tetroxide (Polysciences Inc., USA) and dehydrated in a graded ethanol series (50%, 70%, 90%, 96%, and 100%) and propylene oxide. Propylene oxide to Epon 812 mixtures, at 2:1 (v:v) and 1:2 (v:v), was applied to infiltrate the samples, which were later embedded in Epon 812 epoxy resin (SERVA Electrophoresis, Germany), and polymerized for 48 h at 60 °C. Ultrathin sections were cut from representative samples, using a diamond knife (45°; RMC, USA) on a Reichert OmU-3 ultramicrotome (Reichert Austria). Sections were mounted on 300-mesh copper grids and stained with 0.5% aqueous uranyl acetate and lead citrate (LAURYLAB Saint, France), using a Leica EM AC 20 Stainer (Leica Microsystems, Austria). The grids were air-dried and later examined with a TECNAI G2 12 Spirit BioTWIN transmission electron microscope (FEI, the Netherlands), at 120 kV. The Morada CCD camera (Olympus Soft Imaging System Solutions GmbH, Germany) was used to capture images (tif format, 8-bit, 2656 × 3360 pixels, with 1.5 s integration time, camera binning 1) from representative regions using TEM Imaging and Analysis software (TIA software, version 3.1) (FEI, the Netherlands) in magnification range from 16,500× to 43,000× (Sawczyn et al. 2018).

### Sampling procedure

A researcher blinded to condition chose three to five ultrathin sections from the single mesh grid and photographed capillaries. To avoid bias of double measurements of a single capillary, some sections were discarded between those collected and placed on mesh grid. The researcher used a software tracking tool to record and control move within sections on the grid. The researcher photographed tissue samples within the basal temporal lobe and adjacent to clotted blood for later analysis. A capillary lumen met the inclusion criteria when it was lined with no more than two endothelial cells, surrounded by a basement membrane, cut in cross-section, and did not contain blood cells or plasma. Included samples were photographed under 6000× magnification, to obtain a general view of the capillary and surrounding neuropil, under 11,500× magnification for quantitative analysis, and under 43,000× magnification to identify specific structural traits. Images with an endothelial or pericyte nucleus were excluded from the quantitative analysis because they generated extreme measurements (e.g., for endothelial or pericyte area/coverage/length adhesion) and were too infrequent to adequately sample (Nahirney et al. 2016).

### Morphometric analysis

Given the lack of quantitative analysis that has been performed for capillary ultrastructure in the brain after SAH, we applied a methodological approach of sampling and morphometric analysis that was previously employed to study ultrastructural changes in the brain after ischemia. For quantitative analysis, the ultrastructural traits of the capillary and perivascular area were thresholded and measured using ImageJ software, by an experimenter blinded to condition (Schneider et al. 2012). Other qualitative ultrastructural traits that were observed in the electron micrographs following SAH induction but were difficult to measure were described and compared with capillary profiles in the control (SHAM) and minocycline groups (SAH + Mino).

The ImageJ software functions were used to measure the circularity and roundness of the abluminal and luminal endothelium membranes (capillary and lumen, respectively), where ***circularity*** $\left(4\pi \frac{\mathrm{area}}{{\mathrm{perimeter}}^{2}}\right)$ ranged from 0 (infinitely elongated polygon) to 1 (perfect circle) (Haley and Lawrence 2017), and roundness $\left(4\frac{\mathrm{area}}{\pi *{\left(\mathrm{Major axis}\right)}^{2}}\right)$ was the inverse aspect ratio of the particle-fitted ellipse (Schneider et al. 2012). Then, the capillary and lumen areas were manually outlined in every electron micrograph, to calculate the ratio of endothelium volume fraction $\left(\frac{\mathrm{endothelium\, area}}{\mathrm{capillary\, area}}\right)$ and lumen volume fraction $\left(\frac{\mathrm{lumen\, area}}{\mathrm{capillary\, area}}\right);$; hence, the *endothelium area* was obtained by subtracting the lumen area from the capillary area. These parameters were used to determine changes in the volumes occupied by the endothelium or lumen within the studied capillaries. The capillary area/circumference and lumen area/circumference data were used to calculate the ratios of lumen size $\left(\frac{\mathrm{lumen\, area}}{\mathrm{lumen\, circumference}}\right)$ and capillary size $\left(\frac{\mathrm{capillry\, area}}{\mathrm{capillary\, circumference}}\right)$, to account for variations in the capillary and lumen sizes. The grid overlay image analysis (GOIA) technique was applied to measure the *basal lamina (BL) thickness* (Baum and Bigler 2016). A 0.5 µm × 0.5 µm grid was placed on to capillary electron micrograph, and a minimum of three spots where the gridline crossed the BL was used to calculate the average value of the BL thickness. Places, where the grid crossed the BL adjacent to a pericyte were excluded from analysis because this location is much thinner (Frank et al. 1987; Alba et al. 2004). TJ complexity (*TJ tortuosity*) was measured, as previously described (Jackman et al. 2013), where TJ tortuosity was calculated by dividing TJ length by the diagonal of the rectangle that contains the complete TJ, from where it starts at the luminal side to where it ends at BL. Pinocytic vesicle density was defined as the accumulated number of vesicles per square micrometer, counted in three to five selected fields of the endothelial cytoplasm of one capillary and added together to obtain the reference area (Alba et al. 2004). The structure under study was outlined to measure the pericyte processes area, and when more than one pericyte was present on the profile of a capillary, their areas were added together (Frank et al. 1987). The pericyte processes area was divided by the capillary area to determine the pericyte volume fraction, which represents the volume occupied by pericyte processes inside the capillary wall. The ratio of pericyte or astrocyte coverage was quantified by tracing the pericyte length adhesion or astrocyte length adhesion, including processes, in contact with the endothelial BL and dividing the sum length by the circumference of the BL (Alba et al. 2004; Nahirney et al. 2016). In addition to pericytes, the perivascular space contains astrocyte endfeet. In the majority of electron micrographs from the SAH and SAH + Mino groups, the astrocyte endfoot area was impossible to define because the space adjacent to the capillary was deteriorated; therefore, we used the ImageJ threshold function to measure unequivocally bright spaces adjacent to capillary (the electron-lucent area), which were later called “Holes.” Means from multiple regions of interest (ROI) from one tissue section were used to calculate mean value for each parameter per one animal, used for later analysis.

### Immunofluorescence

Animals (*n* = 3–4 per group) were transcardially perfused with 100 mL ice-cold PBS, pH 7.4, followed by 200 mL 4% paraformaldehyde solution (Merck, Sigma-Aldrich, Germany), and 100 mL 4% paraformaldehyde, with 10% sucrose (Sigma-Aldrich). Brains were quickly removed and post-fixed in 4% paraformaldehyde, with 10% sucrose, overnight at 4 °C. Subsequently, brains were washed in PBS, pH 7.4, and later dehydrated in a graded sucrose series (10%, 20%, and 30%), in PBS, pH 7.4 (weight/volume), until complete dehydration was achieved. Fixed and cryoprotected brains were embedded in TissueTek (Sakura, California, USA) and coronally cryosectioned into 30-µm-thick slices. Collected sections were washed in PBS, pH 7.4, and preserved in PBS, pH 7.4, with 0.05% sodium azide, at 4 °C, for further triple immunostaining procedures on free-floating tissue sections. The staining procedure was performed on an orbital shaker. Briefly, washed sections were pretreated with PBS, pH 7.4, containing 0.1% Triton X-100 (Sigma-Aldrich) for 2 h and then blocked in 10% serum, with 1% Triton X-100, for 1 h. Next, sections were washed in PBS, pH 7.4, and incubated at 4 °C overnight with primary antibodies diluted in PBS, pH 7.4, containing 1% serum and 0.1% Triton X-100. The following antibodies were used (manufacturers provided proof of validation on the technical specification insert): EMMPRIN (1:50, mouse, Bio-Rad, USA), EMMPRIN (1:100, rabbit, Santa-Cruz, USA), collagen IV (1:50, donkey, Bio-Rad), occludin (1:100, rabbit, Antibodies-online, Germany), Pan-Laminin (1:200, rabbit, Sigma-Aldrich), claudin-5 (1:100, rabbit, Antibodies-online), MMP-9 (1:200, rabbit, Abcam, UK), MMP-2 (1:200, rabbit, Abcam), GFAP (1:200, rabbit, Abcam), MAP-2 (1:200, mouse, Invitrogen, USA), AIF-1/Iba-1 (1:200, Antibodies Online), and CD-45 (Novius Biologicals, USA). Antibodies were carefully chosen to avoid cross-reactivity in triple immunofluorescence staining. After washing with PBS, pH 7.4, containing 0.1% Triton X-100 (4 × 5 min), sections were incubated for 1 h with three different secondary antibodies (conjugated with fluorochrome) simultaneously: DyLight conjugated with goat anti-donkey IgG (1:1,000, Jackson Immunoresearch, UK), Alexa 488 conjugated with goat anti-rabbit IgG (1:1,000, Jackson Immunoresearch), and tetramethylrhodamine conjugated with goat anti-mouse IgG (TRITC, 1:1,000, Jackson Immunoresearch). Gentle orbital shaking was used during fixation and staining procedures. After staining, sections were mounted on a microscope slide, after careful washing with PBS, pH 7.4, containing 0.1% Triton-X 100, and PBS, pH 7.4, covered with Vectashield Mounting Medium (Vector Laboratories, USA), and closed with coverslips. Potential unspecific binding of secondary antibodies was evaluated by omission of the primary antibody in sections that were otherwise processed similarly. Sections were stored at 4 °C, in the dark, until further viewing on a confocal microscope.

### Confocal microscopy image acquisition

The sections were photographed under Olympus Fluoview FV1000 confocal microscope, (Olympus Europa Holding, Germany), equipped with Olympus FV1000 SIM scanner system (Olympus Europa Holding) using FV10-ASW 4.2a acquisition software (Olympus Europa Holding), with the following argon lasers: 405 nm, 488 nm, and 559 nm and objective lens: magnification 30; NA 1.05; super apochromat; model UPLSAPO3XS. Additional image acquisition information: PMT voltage 140–160; voxel size 0.414 µm *XY*, 0.82 µM *Z*; resolution 1024 × 1024; image bit depth 12 bit. Three different regions within the basal temporal lobe that were adjacent to clotted blood were scanned every 0.2 µm in the *z*-axis, on multiple focal planes, and in three channels simultaneously, to perform three-dimensional (3D) colocalization analysis. Both the immunostaining procedures and image acquisition with confocal microscopy were performed using identical settings for all samples. Therefore, relative comparisons between different samples treated with the same antibody could be performed. Subsequently, confocal *z*-stacks underwent the denoise procedure and the 3D deconvolution procedure in Autoquant X3.1 (Media Cybernetics Inc, USA) and were analyzed in Imaris 9.3 (Bitplane AG, Switzerland). The extent of colocalization between two labels (DyLight and Alexa 488 or TRITC) was measured using a threshold-based colocalization algorithm, as previously described (Raji et al. 2008). The results were presented as colocalization percentages and Pearson’s correlation coefficients (*r*). *r* values equal to or greater than 0.5 were considered as two fluorescent signals correlating between each other, varying in the same voxel. Mean values for percentage of material colocalized and Pearson’s correlation coefficient (*r*) are included in Supplementary Table I and every scatterplot of Supplementary Figs. I–III. The extent of colocalization of two stains was assessed using 3D colocalization and spot detection, implemented in Imaris software, which allowed us to analyze the percentage of material colocalized in whole stacks of confocal sections, as described by Costes et al. (2004).

### Immunoblotting

The animals were transcardially perfused (*n* = 10–16 per group) with 100 mL PBS, pH 7.4, and brain samples were cut and stored for later protein and mRNA expression analyses. Then, 50–60 mg of frozen brain sample was preincubated in the ice-cold lysis buffer for 15 min, pH 7.4 (20 µL/1 mg of tissue), consisting of 20 mM Tris–HCl, 150 mM NaCl, 2 mM 1% Triton X-100, 10% glycerol, 1 mM PMSF, Protease Inhibitor Cocktail, and PhosSTOP (Sigma-Aldrich), in quantities recommended by the manufacturer, then mechanically homogenized and incubated for additional 20 min in ice-cold lysis buffer. Tissue lysates were centrifuged at 12,000g, at 4 °C, for 10 min. The protein concentration was estimated by the Bradford method, using the Protein Assay Kit (Bio-Rad). The samples containing 30 µg per lane were separated by 10% sodium dodecyl sulfate–polyacrylamide gel electrophoresis (SDS–PAGE) (Carl Roth, Germany) and electrotransferred onto a polyvinylidene difluoride Immobilon-FL Transfer Membrane (Merck, Millipore Solutions, Germany). The membranes were rinsed in PBS (pH 7.4) and blocked in PBS Odyssey Blocking Buffer (pH 7.4) (LI-COR Biosciences, USA), for 1 h at room temperature. Next, the membrane was incubated at 4 °C for 24 h with the following primary antibodies diluted in PBS, pH 7.4 Odyssey Blocking Buffer, with 0.02% Tween 20: EMMPRIN (1:500, rabbit, Santa Cruz), occludin (1:2000, rabbit, Antibodies-online), and claudin-5 (1:2000, rabbit, Antibodies-online) (Sigma-Aldrich). Membranes were washed in PBS, pH 7.4, containing 0.01% Tween 20 (4 × 5 min), and then incubated for 1 h with secondary antibody conjugated to IRDye 800 CW (1:15,000, goat, rabbit, LI-COR), diluted in PBS, pH 7.4 Odyssey Blocking Buffer containing 0.02% Tween 20 and 0.01% Triton-X 100. After washing in PBS, pH 7.4, containing 0.01% Tween 20 (4 × 5 min), and then PBS, pH 7.4 (2 × 5 min), the membranes were dried and scanned with the Odyssey CLx Imaging System (LI-COR). Scanned membranes were activated once more in methanol and then incubated in stripping buffer, containing 0.1 M glycine, pH 2.9, for 1 h at room temperature. Immunoblotting for β-actin (1:2,000, rabbit, Cell Signaling, USA), as a loading control, was performed as described above. ImageStudioLite (LI-COR) was used to quantify the fluorescence intensities of each band, adjusted by the membrane background value measured above and beneath each sample, and normalized against the intensity of the loading control. The average values of protein levels in SHAM control samples, on the same membrane as the SAH and SAH + Mino samples, were used to calculate the fold changes in protein levels.

### Quantitative real-time PCR

Following transcardial perfusion, *n* = 10–16 per group, 15–20 mg of the cortex was rapidly frozen on dry ice and protected in RNA later (EURx, Polska) buffer, until the mRNA isolation protocol was performed. mRNA was extracted using the Total RNA Mini kit (A&A Biotechnology, Poland), according to the manufacturer’s recommendations. Reverse transcription (of 100 ng/µL) was performed with the NG dART RT Kit (EURx), in a DNAEngine DNA thermocycler (Bio-Rad). Quantitative real-time PCR was performed to measure basigin 2 (BSG2), occludin, claudin-5, MMP-2, and MMP-9 transcriptional levels using the CFX96TM Real-Time System (Bio-Rad), in default mode, with SYBR Green Master Mix, using following primers (Genomed, Poland): GAPDH, forward 5′-TGGAAAGCTGTGGCGTGAT-3′ and reverse 5′-AACGGATACATTGGGGGTAG-3′; OCLN, forward 5′- TAGCCATTGTCCTGGGGTTCAT-3′ and reverse TTTCTTCGGGTTTTCACAGCAAA-3′; CLDN5, forward 5′TAAGGCACGGGTGGCACTCA-3′ and reverse 5′-CTACGATGTTGGCGAACCAG3′; BSG2, forward 5′-GTTTGTGAAGCTGATCTGCAAG3′ and reverse 5′-ACAGCTCAGGCGTGGATATAAT-3′; MMP-2, forward 3′-GCAACCACAACCAACTACGA-3′ and reverse 5′-CCAGTGTCAGTATCAGCATCAG-3′; MMP-9, forward 5′-GCAAACCCTGCGTATTTCCAT-3′ and reverse 5′-CCATCCGAGCGACCTTTAGTG-3′. mRNA levels were measured relative to glyceraldehyde 3-phosphate dehydrogenase (GAPDH) mRNA levels, which served as an internal control. Relative mRNA levels were calculated using the comparative *C*_T_ method and expressed using the 2^−ΔΔCT^ method, as fold changes relative to control samples (Livak and Schmittgen 2001).

### Gelatin zymography

Protein extraction was performed, as described previously (Wiera et al. 2013), with some modifications. Tissue homogenization was performed in ice-cold 50 mM Tris–Cl buffer, containing 10 mM CaCl_2_, 0.25% Triton X-100, and 0.1 mM PMSF (30 µL buffer per 1 mg wet tissue). Homogenates were centrifuged at 6000*g*, for 30 min at 4 °C, and the pellet containing the Triton X-100-insoluble proteins was then resuspended in 50 mM Tris–Cl buffer, pH 7.4, containing 2% Triton X-100 and 0.1 M CaCl_2_, incubated for 12 min at 60 °C, and centrifuged (30 min, 10,000*g*, 4 °C). The Amicon Ultra-0.5 3 K (Millipore Sigma) column was used to concentrate the supernatant, containing the extracellular matrix and membrane-associated proteins. Protein concentrations were measured using the Protein Assay Kit (Bio-Rad). Equal amounts of proteins were separated, under nonreducing conditions, using an 8% polyacrylamide (Carl Roth) gel that was copolymerized with 2% fish-skin gelatin (Sigma-Aldrich). After electrophoresis, gels were washed in 2.5% Triton X-100 (2 × 30 min) and incubated in enzymatic buffer (50 mM Tris, pH 7.5, 10 mM CaCl_2_, 1 µM ZnCl_2_, 1% Triton X-100, and 0.02% sodium azide), at 37 °C, for 3–5 days, with continuous, gentle mixing in the STUART Orbital Incubator SI50 (Cole-Palmer, UK). The gels were first stained with 0.1% Coomassie Blue G-250 (Carl Roth) for 2 h and then destained with 5% acetic acid, until white proteolytic bands appeared on the blue background, allowing the visualization of the active MMP levels. Additionally, the same samples were resolved in a parallel SDS–PAGE gel (Carl Roth), without gelatin, to verify protein loads across samples. The Geldoc1000 system (Bio-Rad) was used to digitally photograph gels, and ImageJ (Schneider et al. 2012) was used to quantify the gelatinolytic activity.

### Statistical analysis

Statistical comparisons were conducted with analysis of variance (ANOVA) test, GraphPad Prism 9. For each comparison between groups, a false discovery rate (FDR) correction was performed. All statistical tests were calculated on the basis of means generated from the number of animals in each group. Differences at *P* < 0.05 were considered to be statistically significant.

## Results

### Minocycline protects brain capillaries from SAH-related vessel collapse

**Fig. 2:**
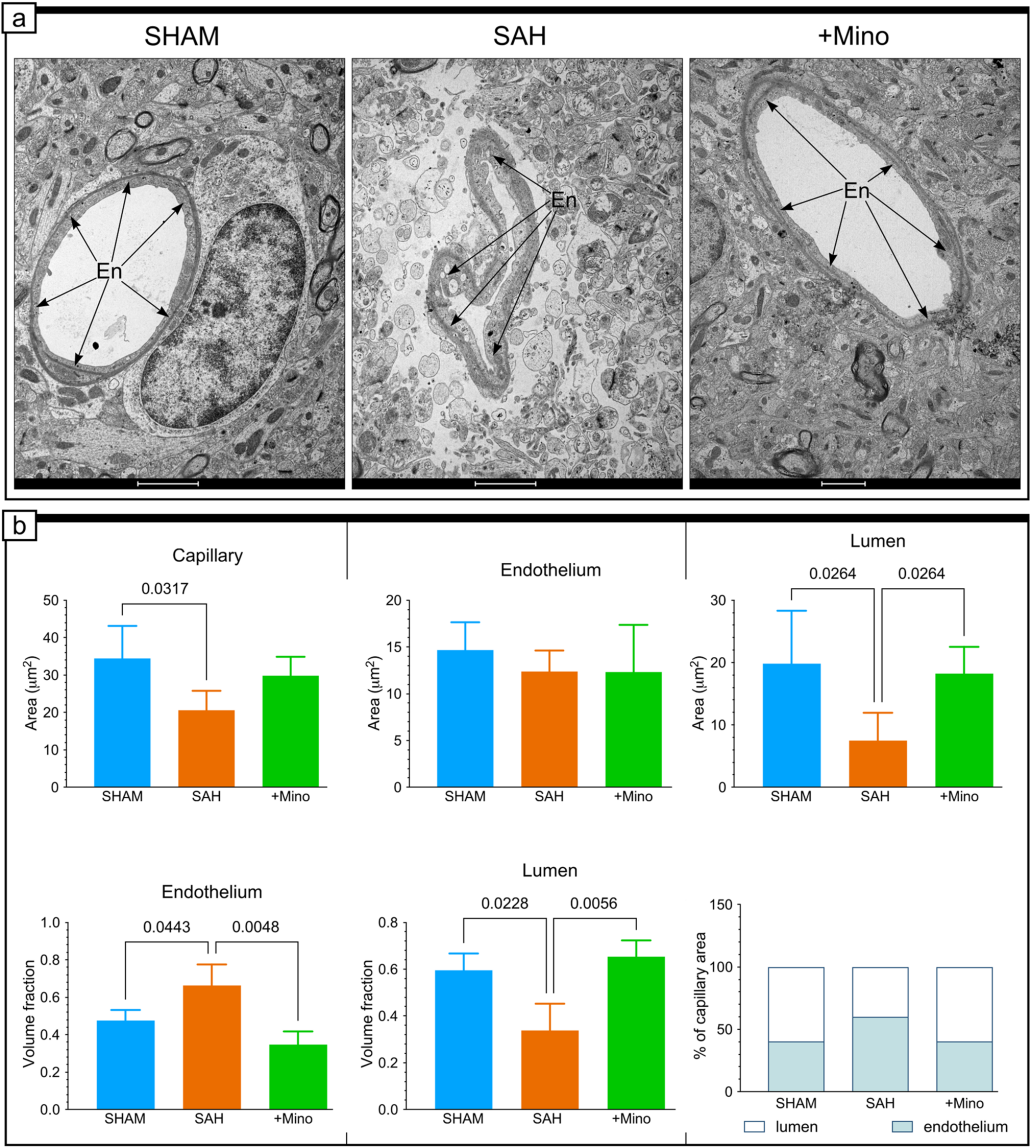
Minocycline protects brain capillaries from SAH-induced changes in vessels. **a** Electron micrographs are representative of vessels from the basal cortex, adjacent to hemorrhage blood, in SHAM, SAH, and SAH + Mino groups. **b** Effect of SAH or SAH and minocycline (1 mg/kg) treatment was quantitatively investigated on the ultrastructural changes in capillaries as a whole, capillary lumen, and capillary endothelium. Data are presented as the mean ± standard deviation (SD), *n* = 3–4 per group. *P* values less than or equal to 0.05 are included in the graphs. See Table 1 for ultrastructural morphometry analysis details. Labeled structures include (En) endothelium (black arrows). Scale bars, 2 μm (**a**)

Animals treated as shown in Fig. 1 were used for the qualitative and the quantitative assessment of the endothelial ultrastructure 24 h after SAH induction. Analysis revealed that capillaries lost their circular shapes, and the luminal and abluminal membrane contours become irregular and began to point toward the capillary lumen (Fig. 2a). Quantitative measurements revealed significant decreases in the capillary area and the lumen area (Fig. 2b), which manifested as increased endothelial volume fractions and reduced lumen volume fractions (Fig. 2b). These results showed that capillaries were more likely to be collapsed than contracted in the SAH group. However, the roundness and circularity of the inner and outer membrane did not show significant changes after SAH. Minocycline had a significant influence on the ultrastructure of tested capillaries, and the results obtained in this group were comparable to those in the SHAM group (Fig. 2a, b).

**Table 1:** Quantification of vessel characteristics from electron micrographs

	SHAM		SAH		SAH + Mino	
	Mean	± SD	Mean	± SD	Mean	± SD
Capillary area (µm^2^)	34.45	8.67	20.58*	5.25	29.86	5.036
Lumen area (µm^2^)	19.79	8.52	7.52*	4.44	18.02***	4.33
Endothelium area (µm^2^)	14.66	3.0	12.37	2.26	12.3	5.06
Lumen volume fraction	0.6	0.07	0.3*	0.1	0.65***	0.06
Endothelium volume fraction	0.48	0.05	0.66*	0.1	0.34***	0.06
Basal lamina thickness (µm)	0.1	0.002	0.1	0.02	0.1	0.07
Tight junction tortuosity	1.25	0.16	1.25	0.08	1.05	0.04
Pericyte area (µm^2^)	5.56	0.98	1.8*	0.4	5.17***	1.67
Pericyte adhesion (µm)	7.43	1.07	5.68	0.2	7.93	1.8
Astrocyte adhesion (µm)	16.8	2.04	7.7*	3.49	13.2***	2.58
Electron-lucent area, hole (µm^2^)	0		22.29*	4.18	0***	
Pinocytic vesicle (no./µm2)	7.48	0.25	4.67*	0.77	8.1***	0.84

Data are presented as mean ± SD, *n* = 3–5 per group

*P < 0.05 SAH versus SHAM, **P ≤ 0.05 SAH + Mino versus SHAM, ***P < 0.05 SAH + Mino versus SAH

### Structural changes in basal lamina morphology do not occur after SAH treatment with minocycline

**Fig. 3:**
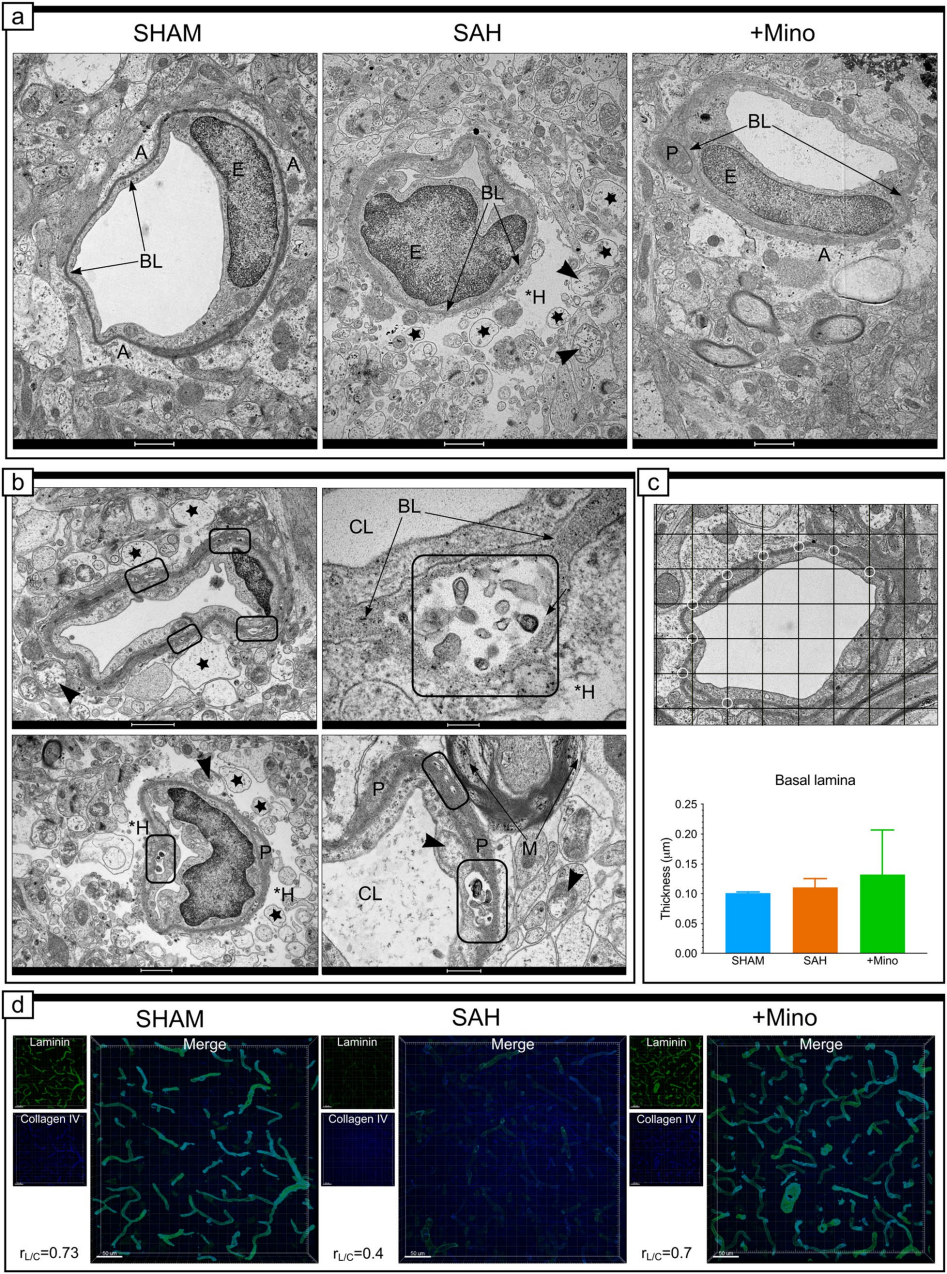
Structural changes in basal lamina morphology do not occur after SAH treatment with minocycline. **a** Representative electron micrograph of vessels and surrounding basal lamina from the basal cortex adjacent to hemorrhage blood of the SHAM, SAH, and SAH + Mino groups. **b** Effect of SAH on ultrastructural changes of basal lamina. **c** Grid was drawn on electron micrographs, and the basal lamina thickness was measured where gridlines crossed within this structure, white circles (GOIA method). *CL* capillary, *A* astrocyte, *H hole, *E* endothelium, *BL* basal lamina, *P* pericyte. Black arrows point to basal lamina. Black arrowheads in the SAH groups point to swollen mitochondria with reduced cristae present within different cells of the neuropil. Stars in the SAH groups point to the vacuole-like structures within degraded areas in the perivascular space. Examples selected in rectangles in the SAH groups point to frequent segments of basal lamina delamination and/or detachments from the endothelium, with accumulations of electron-dense material. Data are presented as mean ± SD, *n* = 3–4 per group. *P* values less than or equal to 0.05 are included in the graphs. See Table 1 for ultrastructural morphometry analysis details. **d** Confocal micrographs showing double immunostaining for laminin (green) and collagen IV (blue), representative of the SHAM, SAH, and SAH + Mino basal cortex adjacent to hemorrhage blood. Single-channel confocal fluorescent micrographs for laminin and collagen IV are located on the left side, next to the large merged image in each panel with PCC value for each group. Data are presented as the mean, *n* = 3–4 per group. Scale bar, 50 µm. See Supplementary Fig. IC and Supplementary Table I for colocalization analysis details. Scale bars, 1 μm (**a**;** b**, top and bottom left), 200 nm (**b**, top right), 500 nm (**b**, bottom right), and 10 μm (**d**)

The capillaries and pericytes in the SHAM group were encircled by a thin layer of BL, which, together with the endothelium and astrocytic endfeet, created numerous and dense contacts (Fig. 3a, SHAM). After SAH, the BL contour became irregular (Fig. 3a, SAH), and along the perimeter of the capillary, we noted numerous sections of different lengths and locations where the BL was delaminated or detached from the endothelial cell (Fig. 3b). Within these subendothelial spaces, electron-dense cellular fragments/processes were identified (Fig. 3b). The outer layer of the BL, surrounding pericytes, was wrinkled, with irregular invaginations toward the neuropil. However, analysis showed constant BL thickness at measured points along the perimeters of tested capillaries, regardless of the experimental group (Fig. 3c).

Additionally, we investigated the effects of SAH on laminin and collagen IV proteins (Fig. 3d), to determine whether the in situ changes to these proteins reflected the ultrastructural abnormalities in the BL. The high percentage of colocalized material (%CM) indicated the strong and widespread colocalization of laminin and collagen IV, and the high Pearson’s correlation coefficient (PCC) between the colocalized voxels showed that these proteins varied together in the SHAM group (Fig. 3d, SHAM). The PCC for laminin and collagen IV decreased in the SAH samples, owing to changes in the laminin fluorescent content (Fig. 3d, SAH). Analysis of the electron micrographs and immunofluorescent images obtained for the SAH + Mino group showed that minocycline administration in rats effectively protected the BL from the morphological abnormalities described for the SAH group (Fig. 3a SAH, 3b), and the laminin/collagen IV colocalization parameters remained similar to those observed for the SHAM group (Fig. 3d, +Mino).

### Tight junctions retained their ultrastructure, despite subarachnoid hemorrhage induction

**Fig. 4:**
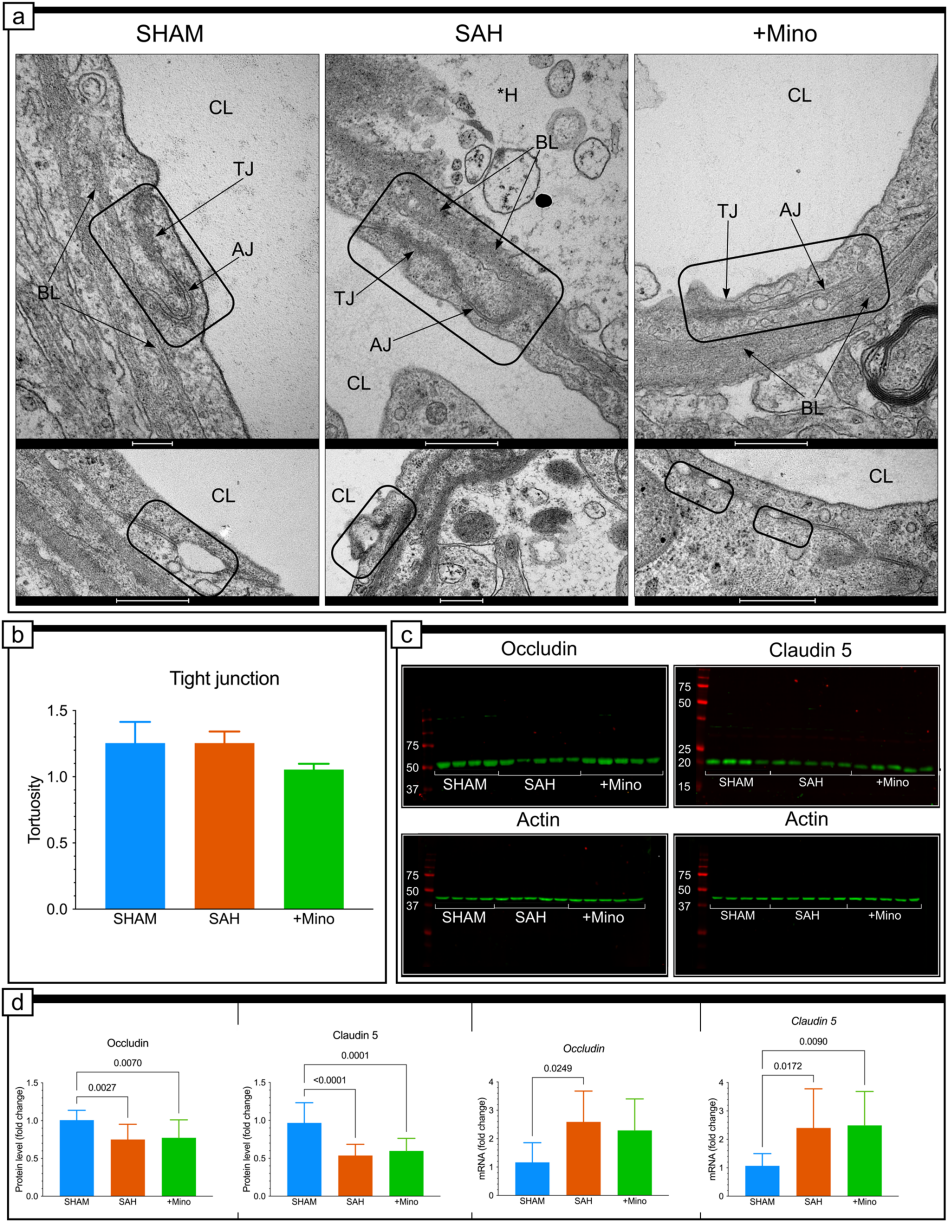
Tight junction ultrastructure, and occludin or claudin-5 protein levels and gene expressions. **a** Representative electron micrograph of tight junctions between endothelial cells of capillaries from basal cortex adjacent to hemorrhage blood for the SHAM, SAH, and SAH + Mino groups. Rectangles in top micrographs point to junctional morphology for each group, when examples selected in rectangles on the bottom micrographs show rare cases of short openings along junctional connections between two adjacent endothelial cells in SAH group. Black arrows point to basal lamina. *CL* capillary, *TJ* tight unction, *AJ* adherence junction, *BL* basal lamina, *H hole. **b** Effect of SAH or SAH and minocycline (1 mg/kg) treatment was quantitatively investigated on the ultrastructural changes of tight junctions tortuosity. Data are presented as mean ± SD, *n* = 3–4 per group. *P* values less than or equal to 0.05 are included in the graphs. See Table 1 for ultrastructural morphometry analysis details. **c** Fluorescence representation of both occludin and claudin-5 and control β-actin immunoblotting. **d** Effect of SAH or SAH and minocycline treatment on occludin and claudin 5 protein level, and *occludin* and *claudin 5* gene expression. Data are presented as mean ± SD, *n* = 10–16 per group. *P* values less than or equal to 0.05 are included in the graph. Scale bar, 500 nm (**a**)

TJs are characterized by a tortuous overlap between adjacent endothelial cells, which appear as areas of close apposition between neighboring membranes, with a fusion of the outer membrane leaflet (Fig. 4a, SHAM). The protein levels of occludin and claudin-5 decreased significantly in brain tissues (Fig. 4c, d). However, we did not observe any visible morphological changes to the TJ ultrastructure, although small, fluid-filled spaces were observed in no more than 8% of all tested TJs in SAH group (Fig. 4a, bottom panels). TJs maintained electron-dense interdigitating structures between two consecutive endothelial cells, even in severely transformed vessels, following the degenerative influence of SAH on neurovascular unit (NVU) morphologies (Fig. 4a, SAH). Analysis revealed no differences in TJ tortuosity between groups (Fig. 4b). The expression levels of *claudin-5* mRNA were upregulated in minocycline rats compared with SHAM rats (Fig. 4d); however, minocycline administration had no effects on occludin and claudin-5 protein levels (Fig. 4d). *Occludin* and *claudin-5* mRNA expression was upregulated in the SAH group compared with controls (Fig. 4d).

We also explored the localization of occludin and claudin-5 in the interendothelial junctions of capillaries. The colocalization analysis (CA) results indicated limited and weak colocalization between occludin and collagen IV in the SHAM group (Supplementary Fig. Ia). The colocalization between claudin-5 and collagen IV was similar to that for the occludin/collagen IV analysis; however, claudin-5 and collagen IV varied together when present in the same voxel (Supplementary Fig. Ib). When SAH was initiated, the colocalization increased for both analyses (Supplementary Fig. Ib). According to the immunofluorescence pattern, the green fluorescence signal of occludin was stronger than the collagen IV blue signal, and only a small population of occludin colocalized with collagen IV (Supplementary Fig. Ia). In contrast, the analysis examining claudin-5 and collagen IV revealed a high degree of colocalization and showed that these two proteins were correlated (Supplementary Fig. Ia). After minocycline administration, the scale of colocalization visibly decreased for both occludin/collagen IV and claudin 5/collagen IV (Supplementary Fig. Ia and Ib).

We also investigated the colocalization between EMMPRIN and occludin or claudin-5. The level of colocalization and correlation assessed for the SHAM group indicated no relationship between EMMPRIN and occludin or between EMMPRIN and claudin-5 (Supplementary Fig. Ia and Ib). When SAH was initiated, we observed elevated colocalization between EMMPRIN and both occludin and claudin-5 and slightly increased correlations between them (Supplementary Fig. Ia and Ib). The administration of minocycline did not influence the changes observed in the SAH group (Supplementary Fig. Ia and Ib).

### Minocycline prevents ultrastructural changes in pericytes

**Fig. 5:**
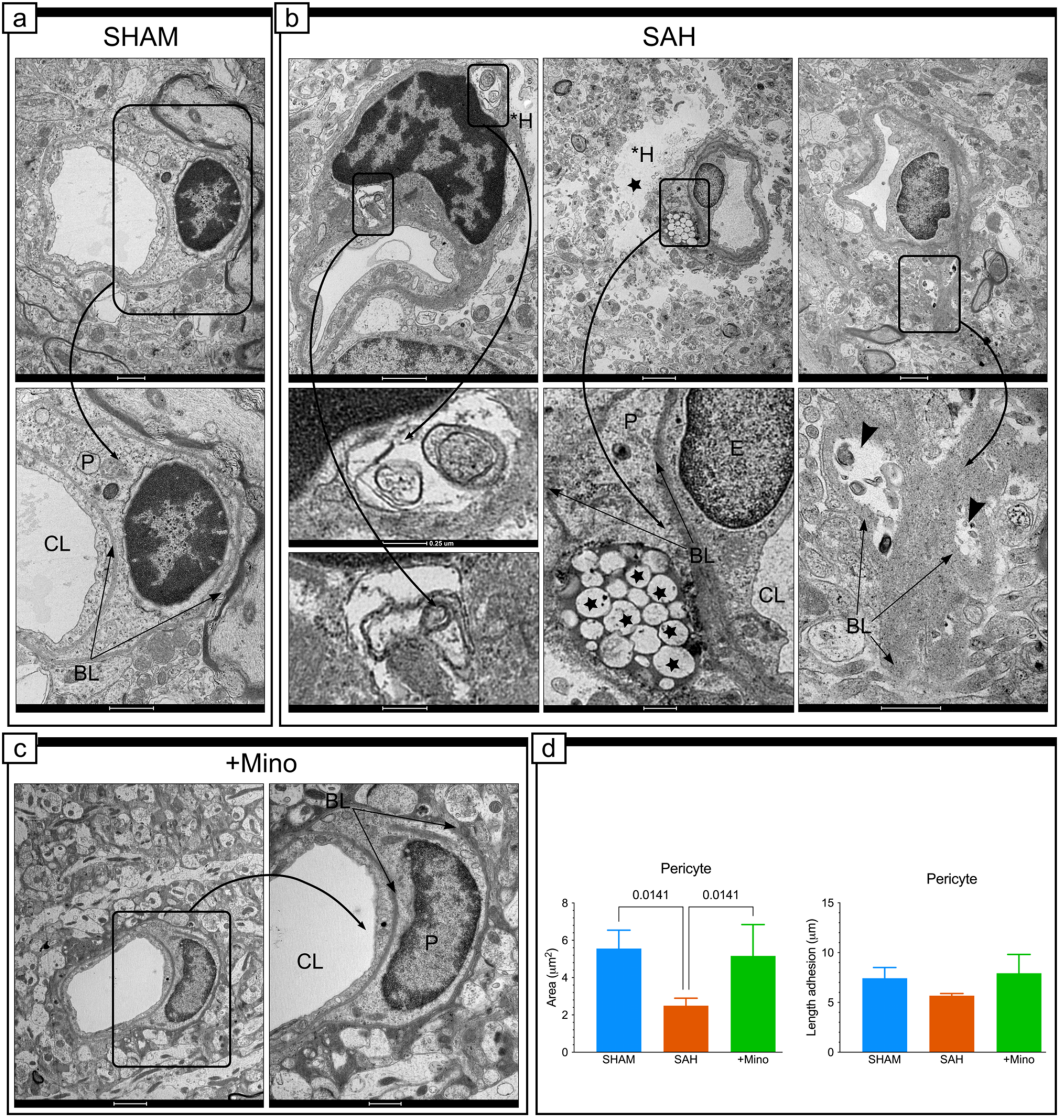
Minocycline prevents ultrastructural changes in pericytes. **a** Representative electron micrograph of the vessels and adjacent pericytes in the SHAM group. The area enclosed by the rectangle reproduced at higher magnification (inset) shows representative pericyte morphology in the SHAM group. **b** Effect of SAH on the pericyte morphology. Selected areas enclosed by the rectangle show: autophagosomes present in the cytoplasm of a pericyte adjacent to a capillary (**b**: left micrographs), visible collection of lipid droplets in the cytoplasm of the pericyte (**b**: middle micrographs), and ruffled basal membrane that remains after the retraction of a pericyte process (**b**: right micrographs). Arrowheads point to deposits of cellular debris. Black star and *H point to presence of the electron-lucent space in the perivascular area surrounding the capillary. **c** Effect of SAH and minocycline treatment on pericytes. The area enclosed by the rectangle reproduced in the inset shows representative pericyte morphology in the SAH + Mino group. *CL* capillary, *H hole, *E* endothelium, *BL* basal lamina, *P* pericyte. Black arrows point to basal lamina. **d** Result of SAH or SAH and minocycline treatment on quantitative parameters. Data are presented as mean ± SD, *n* = 3–4 per group. *P* values less than or equal to 0.05 are included in the graph. See Table 1 for ultrastructural morphometry analysis details. Scale bars, 1 μm (**a**; **b**, top left and right), 2 μm (**b**, top middle; **c**), 250 nm (**b**, bottom left), 500 nm (**b**, bottom middle and right)

Qualitative analysis revealed that pericytes from SAH-affected capillaries often displayed traits associated with two typical types of transformation. Pericytes displaying shrunken somas and processes and filled with electron-dense lumps, which appeared to be cellular debris, were categorized as degenerating cells (Fig. 5b). In contrast, pericytes displaying swollen somas and processes, in which the cytoplasm contained dense lysosomal bodies and lipid-like droplets of different sizes (Fig. 5b), sometimes accompanied by autophagosomes, were categorized as activated and transforming cells (Fig. 5b). The quantitative analysis of the pericytes showed significant decrease in their area in response to SAH induction (Fig. 5d), without changes in adhesion length to the BL (Fig. 5d). The pericyte area in minocycline-injected rats significantly increased (Fig. 5d).

### MMP-2 but not MMP-9 or EMMPRIN play significant role in the low-dose minocycline neuroprotective actions following SAH

**Fig. 6:**
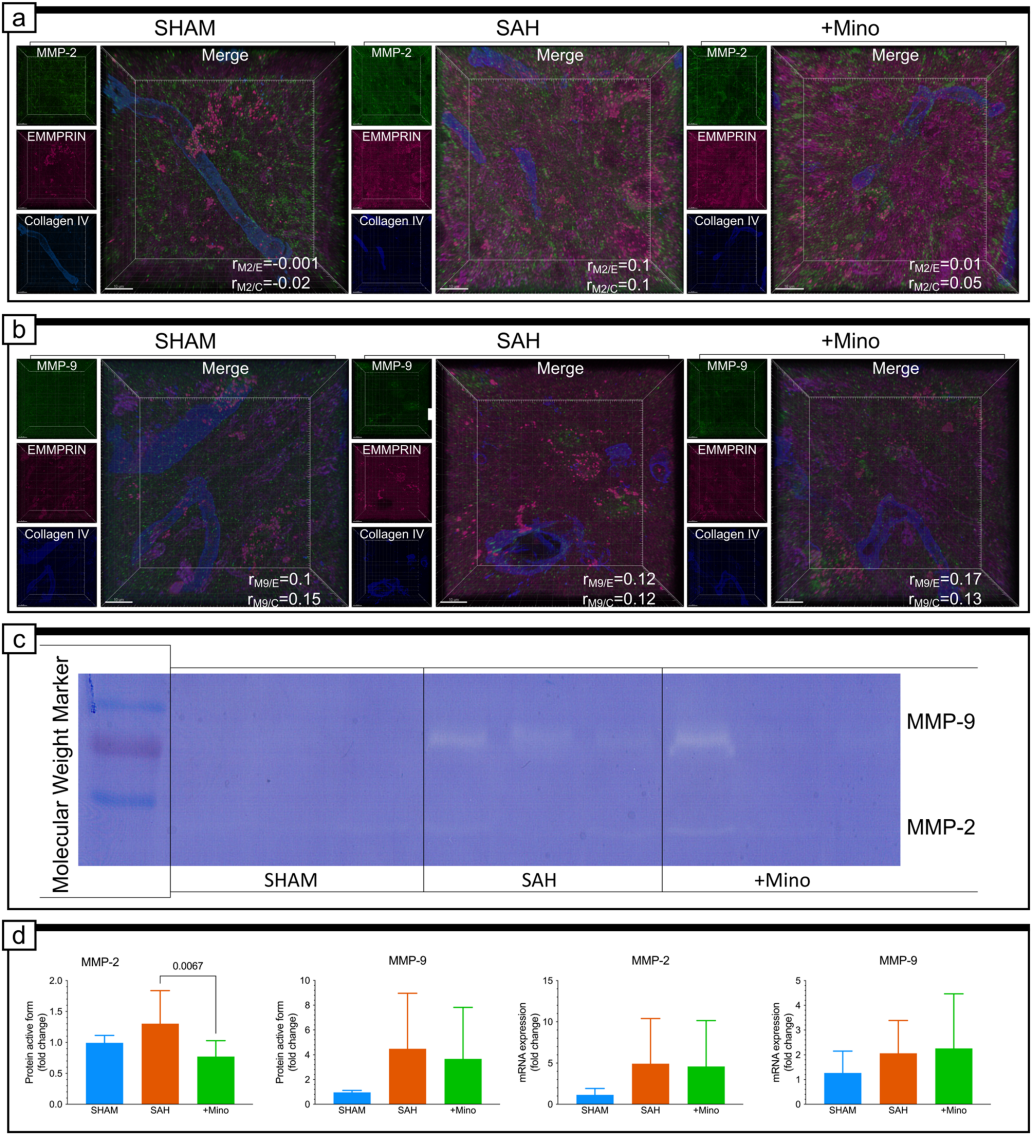
MMP-2, MMP-9 colocalization with EMMPRIN or collagen IV, protein levels and gene expressions. **a** Representative confocal micrograph showing merged picture of triple immunostaining for MMP-2 (green), EMMPRIN (magenta), and collagen IV (blue) and single-channel pictures situated on their left side for each panel of the SHAM, SAH, and SAH + Mino group. Pearson’s correlation coefficient value included in the panel: (*r*_M2/E_) for MMP-2 and EMMPRIN, and (*r*_M2/C_) for MMP-2 and collagen IV. **b** Representative confocal micrographs showing merged picture of triple immunostaining for MMP-9 (green), EMMPRIN (magenta), and collagen IV (blue) and single-channel pictures situated on their left side for each panel of the SHAM, SAH, and SAH + Mino group. Parameter (*r*_M9/E_) reflects value of correlation between MMP-9 and EMMPRIN fluorescent signals as well as (*r*_M9/C_) to MMP-9 and collagen IV. Data are presented as the mean, *n* = 3–4 per group. Scale bar, 10 µm. See Supplementary Fig. IIB, IIC and Supplementary Table I for colocalization analysis details. **c** Representative picture of gelatin zymography. **d** Effect of SAH or SAH and minocycline treatment on MMP-2 and MMP-9 protein active form level and their gene expression. Data for protein active form (*n* = 8–16) and gene expression (*n* = 10–14) are presented as mean ± SD. *P* values less than or equal to 0.05 are included in the graph. Scale bar, 10 μm (**a**, **b**)

The percentage of material colocalized (%MC) showed the widespread colocalization of MMP-2/collagen IV, but they did not vary together in any groups (Fig. 6a and Supplementary Fig. IIb). Similar results were obtained for the MMP-9/collagen IV colocalization analysis (Fig. 5b and Supplementary Fig. IIc). Next, we studied the levels of the enzymatically active forms of MMP-2 and MMP-9 and their mRNA expression levels in the same tissue samples, collected from brain areas adjacent to hemorrhagic blood. Statistical analysis showed significant differences in MMP-2 protein levels between SAH + Mino and SAH group, but no changes in mRNA expression levels between groups (Fig. 6d). MMP-9 gelatin zymography revealed no significant increase of protein active form both in SAH and SAH + Mino groups compared with control (Fig. 6d). No change in *MMP-9* gene expression was observed between the groups (Fig. 6d).

**Fig. 7:**
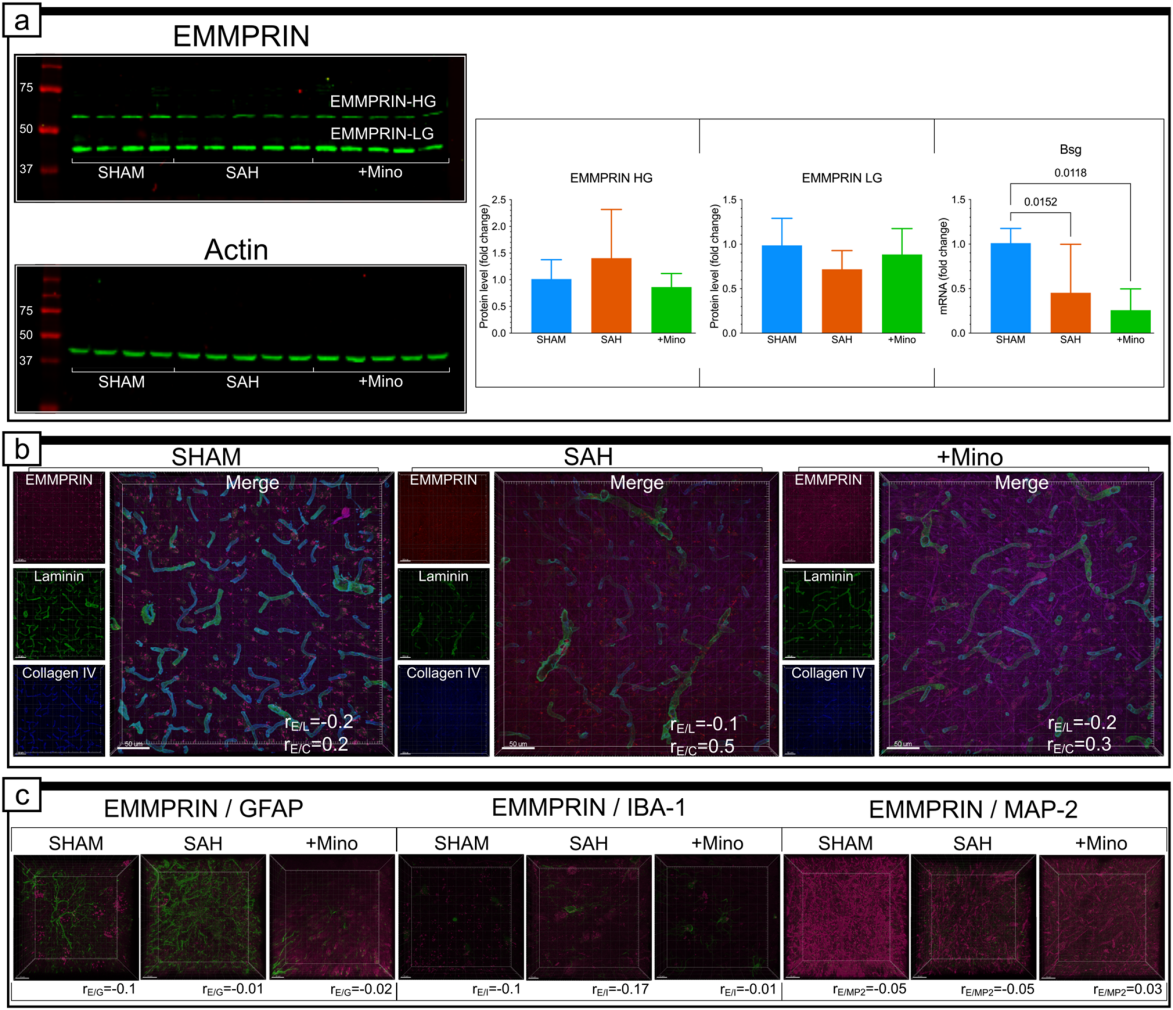
EMMPRIN protein level, gene expression, and colocalization with laminin, collagen IV, GFAP, IBA-1, and MAP-2. **a** Fluorescence representation of EMMPRIN and β-actin immunoblotting and SAH or SAH and minocycline treatment on highly glycosylated form (EMMPRIN-HG) and the low-glycosylated form (EMMPRIN-LG), and *Bsg* gene expression. Data are presented as mean ± SD *n* = 10–16 per group for protein level and *n* = 10–14 per group for gene expression. *P* values less than or equal to 0.05 are included in the graph. **b** Confocal micrographs showing triple immunostaining for laminin (green), EMMPRIN (magenta), and collagen IV (blue) and single-channel pictures situated on their left side for each panel of the SHAM, SAH, and SAH + Mino group. Parameter (*r*_E/L_) reflects value of correlation between EMMPRIN and Laminin fluorescent signals as well as (*r*_E/C_) to EMMPRIN and collagen IV. Data are presented as the mean, *n* = 3–4 per group. See Supplementary Fig. IIIA and Supplementary Table I for colocalization analysis details. **c** Colocalization studies performed for EMMPRIN (magenta) and GFAP (green), or IBA-1 (green), and EMMPRIN (green) and MAP-2 (magenta). Pearson’s correlation coefficient value included beneath confocal micrographs: (*r*_E/G_) for EMMPRIN and GFAP, (*r*_E/I_) for EMMPRIN and IBA-1, and (r_E/MP2_) for EMMPRIN and MAP-2. Data are presented as the mean, *n* = 3–4 per group. Scale bars, 50 μm (**a**) and 10 µm (**b**)

We also performed histological and biochemical analyses for EMMPRIN protein, which has been described as performing upstream activation of gelatinases (Papadimitropoulou and Mamalaki 2013; Grass and Toole 2015). The results indicated no correlations between EMMPRIN/MMP-2 or EMMPRIN/MMP-9, despite the high percentage of colocalization observed within the same voxels in every group (Fig. 6a, b and Supplementary Fig. IIb, IIc). However, EMMPRIN was present in the BL of the studied capillaries. In the SHAM tissue samples, EMMPRIN expressed moderate levels of colocalization and a weak correlation with collagen IV (Fig. 7b and Supplementary Fig. IIIa). However, when we assessed the extent of colocalization between these proteins in the SAH group, EMMPRIN presented widespread colocalization and a strong correlation with collagen IV (Fig. 7b and Supplementary Fig. IIIa), even though the levels of weakly and highly glycosylated protein forms were similar between groups (Fig. 7a). In contrast, the *BSG2* mRNA expression level was significantly downregulated by SAH and was insensitive to minocycline administration (Fig. 7a). The only visible effects of minocycline were decreased colocalization and correlation values for EMMPRIN and collagen IV (Fig. 7b). We also explored the EMMPRIN signal we detected in the parenchyma (Fig. 7b), to verify its origin. The results excluded the possibility that EMMPRIN colocalizes with glial fibrillary acidic protein (GFAP), a molecular marker for astrocytes; microtubule-associated protein 2 (MAP-2), a molecular marker for neurons; and ionized calcium binding adaptor molecule 1 (IBA-1), a molecular marker for microglia, in every group (Fig. 7c and Supplementary Fig. IIIb).

We also investigated the colocalization between EMMPRIN and occludin or claudin-5. The level of colocalization and correlation assessed for the SHAM group indicated no relationship between EMMPRIN and occludin or between EMMPRIN and claudin-5 (Supplementary Fig. Ia and Ib). When SAH was initiated, we observed elevated colocalization between EMMPRIN and both occludin and claudin-5 and slightly increased correlations between them (Supplementary Fig. Ia and Ib). The administration of minocycline did not influence the changes observed in the SAH group (Supplementary Fig. Ia and Ib).

### SAH results in abnormal vacuole and vesicle production

**Fig. 8:**
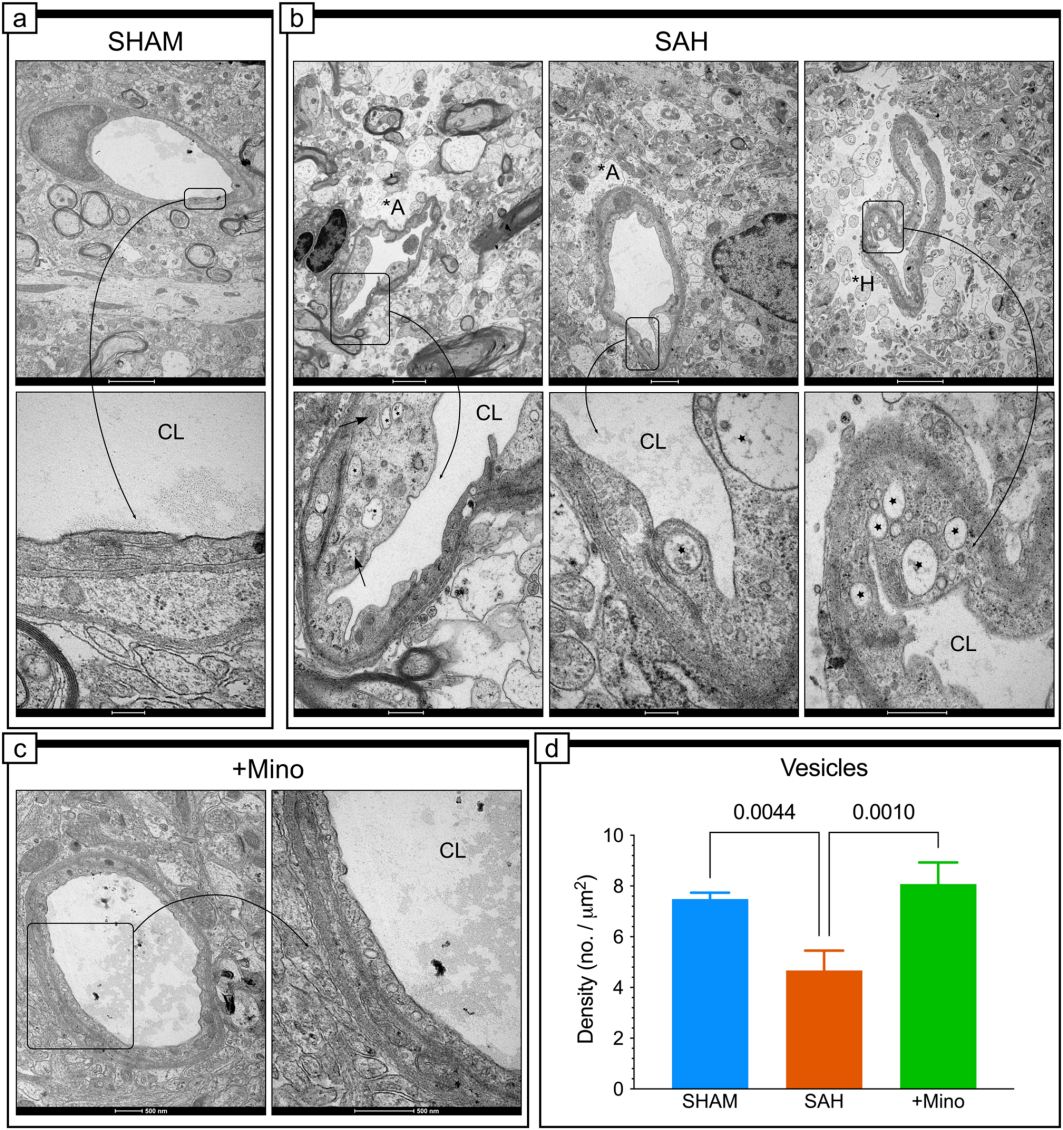
SAH leads to abnormal vacuole and vesicle production. **a** Representative electron micrographs endothelial cells in capillaries in the SHAM group. The area enclosed by the rectangle reproduced at higher magnification (inset) shows representative endothelium morphology in the SHAM group. **b** Effect of SAH on the endothelium morphology. Selected areas enclosed by the rectangle show morphology details of endothelial cell at higher magnification. Stars point to vacuoles of different sizes in various locations in the swollen cytoplasm of the endothelium. Short arrows point to swollen mitochondria with reduced cristae. *A is located where swollen astrocytes are visible, and *H points to presence of the electron-lucent space in the deteriorated perivascular area surrounding the capillary. *CL* capillary, *A swollen astrocyte, *H hole. **c** Effect of minocycline on the endothelium morphology. **d** Effect of SAH and minocycline treatment on vesicle density. Data are presented as mean ± SD, *n* = 3–4 per group. *P* values less than or equal to 0.05 are included in the graph. See Table 1 for ultrastructural morphometry analysis details. Scale bars, 2 μm (**a**, top; **b**, top left and right), 200 nm (**a**, bottom), 1 μm (**b**, top middle), 500 nm (**b**, bottom left and right; **c**), 200 nm (**b**, bottom middle)

A characteristic feature of endothelial cells after SAH was the presence of vacuoles of variable sizes, without any content (Fig. 8b) or containing a granular material (Fig. 8b). These vacuoles could appear at any site within the cell (Fig. 8b), adjacent to TJs (Fig. 8b). The mechanism underlying significant vacuole formation remains unknown but could be related to the previously mentioned long endothelial protrusions (Supplementary Fig. IVc) or marginal folds, which were observed in this study and reported previously (Chiang et al. 1968). These vacuoles could initiate the engulfment of debris from the vessel lumen, such as during microvessel recanalization (Supplementary Fig. IVc) (Chiang et al. 1968; Sehba and Friedrich 2013). However, some of these vacuoles may represent degenerated mitochondria, without crests (Fig. 8b), as we have observed multiple swollen mitochondria containing significantly shortened cristae. Contrary to previous reports, we discovered that the vacuole density was significantly increased in the SAH group compared with the SHAM group (Fig. 8d), and minocycline protected endothelial cells from SAH-induced vacuole production (Fig. 8c) and reversed the pinocytic vesicle density back to control values (Fig. 8d).

### SAH results in far-reaching damage to the neuropil-surrounding capillaries

**Fig. 9:**
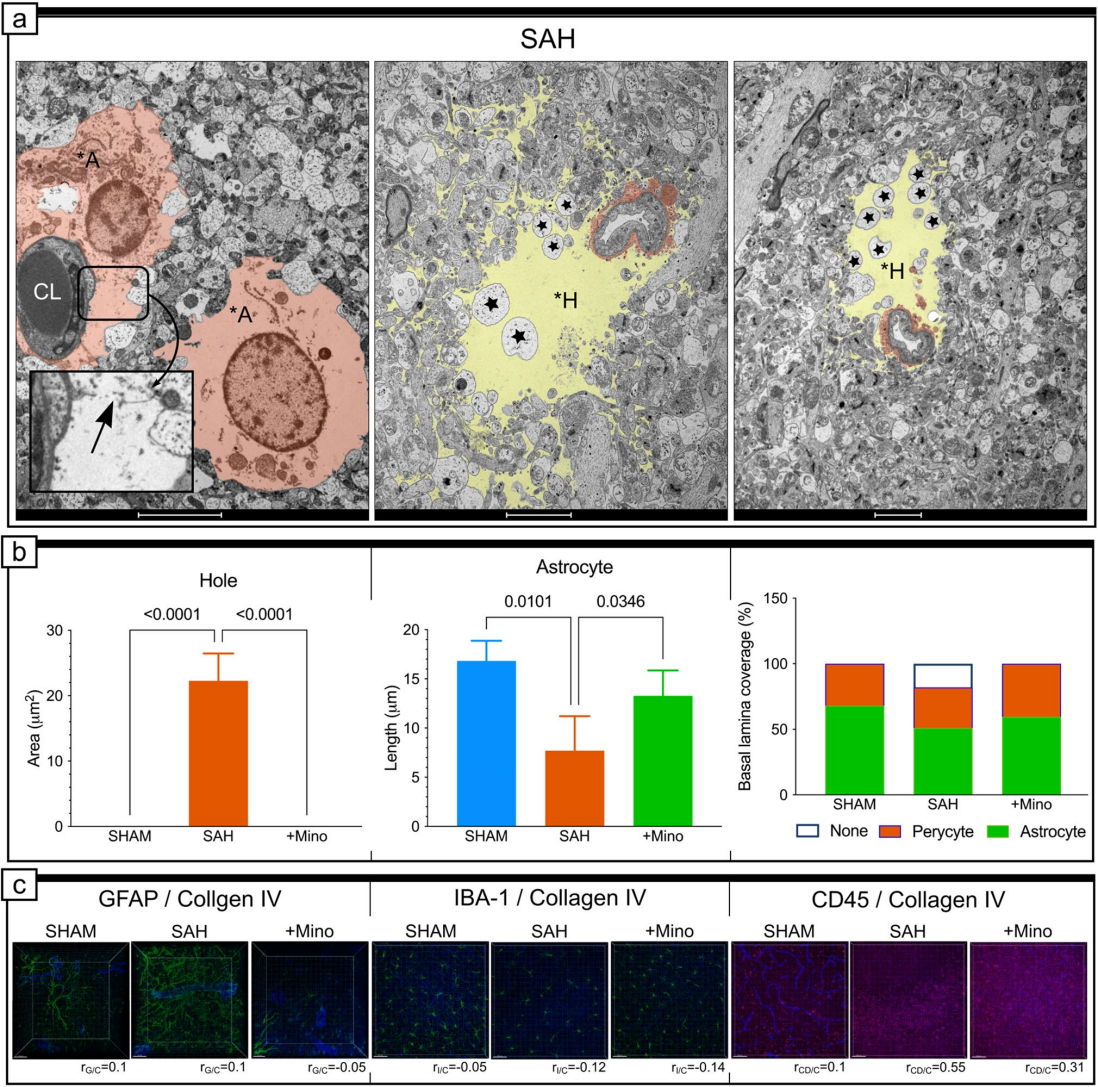
Minocycline protects cells in the perivascular space from degeneration. **a** Representative electron micrograph of capillary and perivascular areas from the basal cortex adjacent to hemorrhage blood for the SAH group. Cells colored in orange show intense astrocyte swelling, with degenerated organelles. The area enclosed by the rectangle, reproduced at a higher magnification, presents disrupted membranes between two astrocytic endfeet. Yellow-colored spaces within micrographs represent deteriorated perivascular area, and astrocytic debris surrounding the capillary is colored in orange. *CL* capillary, *A swollen astrocyte, *H hole. Black stars point to swollen cellular fragments. **b** Effect of SAH or SAH and minocycline treatment on the average size of the deteriorated perivascular area, and astrocyte endfoot length or basal lamina coverage by astrocytes and pericytes. See Supplementary Figs. IV and V for additional examples of the SAH destructive effects on the neuropil. Data are presented as mean ± SD, *n* = 3–4 per group. *P* values less than or equal to 0.05 are included in the graph. See Table 1 for ultrastructural morphometry analysis details. **c** Representative confocal micrographs of double immunostaining for GFAP (green) and collagen IV (blue), IBA-1 (green) and collagen IV (blue), CD-45 (green) and collagen IV (blue). Pearson’s correlation coefficient value included beneath confocal micrographs: (*r*_G/C_) for GFAP and collagen IV, and (*r*_I/C_) for IBA-1 and collagen IV, and (*r*_CD/C_) for CD-45 and collagen IV. See Supplementary Fig. IIA and Supplementary Table I for colocalization analysis details. Data are presented as the mean, *n* = 3–4 per group. Scale bars, 5 μm (**a**, left), 2 μm (**a**, middle and right), and 10 µm (**c**)

The least-affected capillaries were surrounded by deteriorated tissue structure, with swollen astrocyte endfeet containing visible intermediate filaments (Supplementary Fig. Vd) or degenerated organelles, together with disrupted membranes (Fig. 9a). However, far-reaching damage to the perivascular area manifested as electron-lucent areas of various sizes, which were devoid of extracellular matrix or cells and were termed “holes” (Fig. 9a). In an average 20 µm^2^ area (Fig. 9b), we observed swollen cell fragments of irregular sizes within the margins of the “holes” (Fig. 9a). In addition, we noted visible discontinuities in the cell membrane along the border of this area. In view of these observations, we were unable to conduct detailed astrocyte measurements, and the only parameters we were able to calculate were the adhesion length to BL and astrocyte coverage, which significantly decreased after SAH (Fig. 9b). The general picture of GFAP staining showed increased immunoexpression after SAH, and the %MC was high for GFAP and collagen IV (Supplementary Fig. IIa). The GFAP signals were more intense than the collagen IV signals in the same voxels, and the PCC was very low, for every group, indicating no correlation between these proteins (Fig. 9c). We also explored the possible participation of microglia/macrophages (IBA-1) and CD45-positive cells in the development of the significant ultrastructural changes described above. IBA-1 did not colocalize or correlate with collagen IV in any of the studied groups (Fig. 9c). CD45 demonstrated a weak colocalization and correlation with collagen IV in the SHAM group (Fig. 9c). After SAH, the colocalization analysis revealed the large extent of CD45 colocalization with collagen IV and a strong correlation within the same voxel groups (Fig. 9c). Minocycline administration successfully decreased both %MC and PCC values between CD45 and collagen IV (Fig. 9c).

We also reported the distance of capillary morphological changes to the neuropil following SAH initiation (Supplementary Figs. IV and V), especially for neurons compared with the other two groups (Fig. 9a). These cells displayed visible signs of injury, such as affected mitochondria with reduced cristae within dendrites (Supplementary Fig. IVb), synapses (Supplementary Fig. IVb), axons (Supplementary Figs. IVa and Vb), and neuronal cell bodies (Supplementary Fig. Vc), lysosomal dense bodies (Supplementary Fig. Vc), electron-dense cytoplasm and nucleus (Supplementary Fig. Vc), irregular membrane contours (Supplementary Fig. Vc), and flower-like myelin (Supplementary Fig. Vb). Additionally, cross-sections of neuronal fibers contained neurofilaments with segmental loss of continuity and interrupted cell membranes (Supplementary Fig. IVa). We demonstrated that minocycline could prevent the neuropil from being affected by SAH-induced damages (Supplementary Fig. Va, +Mino).

## Discussion

SAH is a complex disease involving several ongoing processes that result in cell death and begin soon after blood is released into subarachnoid space (Chen et al. 2014). Morphological alterations in capillaries play vital roles in the mechanisms involved in functional declines of the CNS. In the current study, the ultrastructural analysis revealed that minocycline administration protected capillaries and surrounding cells from SAH-induced qualitative and quantitative changes, therefore helping to maintain their physiology by protecting their morphology. Proper circulation of blood and its components in brain affected by hemorrhage prevented NVU cells from secondary hypoxia, microthrombosis, and augmented neurodegeneration.

On the basis of the literature survey, we found a paucity of data examining early changes in the NVU ultrastructure following SAH. In general, previous studies using TEM have focused on the assessment of SAH-induced changes in the ultrastructure of large cerebral arteries and factors responsible for later vasospasms and secondary cerebral ischemia (Mayberg et al. 1978; Pickard et al. 1985; Duff et al. 1986; Seifert et al. 1989), whereas small capillaries are more vulnerable to hemorrhagic bleeding (Sehba and Friedrich 2011). The obstruction of small capillaries and the death of surrounding cells are caused by impaired neurovascular coupling and spreading depolarization, which can later propagate to distant areas (Koide et al. 2012, 2013a, b; Balbi et al. 2017). Significant decreases in the average size of capillaries, with enlarged endothelial sizes and reduced lumen sizes, confirmed that capillaries experience more meaningful and rapid compromises in structure and function after SAH compared with large vessels(Sabri et al. 2012; Sehba and Friedrich 2013). The ultrastructural degenerative changes to mitochondria, neurons, and the neuropil in general that were observed in this study have also been observed as characteristic morphological traits associated with diabetic encephalopathy, suggesting that the cells in the brain tissue in SAH were both oxygen- and glucose-deprived (Hernández-Fonseca et al. 2009).

The BL forms a second integrity barrier for the vessel wall, and its degradation has been associated with increased intravascular pressure and increased microvascular permeability, leading to the extravasation of fluid into the brain and edema (Hamann et al. 1995; Sehba et al. 2004; Schöller et al. 2007). The decreased expression of collagen IV and laminins had a visible impact on the microcapillary morphology, which manifested as delaminated and dissociated segments of BL from the endothelium, which are the histological traits for uncontrolled vascular leakage (Bonkowski et al. 2011; Østergaard et al. 2013).

TJs, which seal membranes between opposing endothelial cells, prevent the uncontrolled hydrophilic molecule diffusion into the brain. In contrast, transport vesicles within the endothelial cytoplasm direct BBB selectivity and maintain the blood–brain metabolic concentration gradient (Reese and Karnovsky 1967; Bechmann et al. 2007). TJs were unaffected by SAH, and even stalled or occluded capillaries contained intact TJs. Less than 8% of TJs included subtle perturbations, observed as small spaces at the TJ interfaces, although the majority of TJ were contiguous. These widenings resulted from reductions in occludin and claudin-5 protein levels (Nahirney et al. 2016), which was the effect, but not the cause, of BBB functional loss after SAH (Krueger et al. 2013, 2015).

According to Liu et al., MMP-2, but not MMP-9, is involved in the proteolytic degradation of occludin in TJs after ischemia (Liu et al. 2012), whereas the caveolin-1-related redistribution of claudin-5 into the cell is responsible for the decline in claudin-5 levels (Liu et al. 2012). During the posttranslational modification of EMMPRIN, some immature EMMPRIN monomers interact with caveolin-1, via the immunoglobulin domain, eventually forming membrane-bound heterodimers (Tang et al. 2004). In the present study, we did not find gelatinases to be responsible for occludin level decline; however, weak but positive correlations between EMMPRIN and both occludin and claudin-5 were observed in SAH animals compared with control animals, suggesting that EMMPRIN may be involved in the occludin and claudin-5 declines after SAH induction. Most importantly, the protein loss was supported by significant increases in *occludin* and *claudin-5* gene expression, allowing TJ control of paracellular transport across capillaries to be sustained despite SAH induction.

During acute brain injury, capillary sizes can change through pericyte contraction or astrocytic edema compression (Østergaard et al. 2013). Under the pathological conditions of acute brain injury, pericytes constrict and fold the BL in the region adhering to the endothelium, restricting the capillary lumen and hindering capillary blood perfusion (Sabri et al. 2012; Sehba and Friedrich 2013; Nahirney et al. 2016). No apparent ultrastructural evidence of lumen compression due to circumferential pericyte projections was observed in the present study. However, our observations of pericyte morphologies after SAH demonstrated some qualitative changes compared with control samples. Both forms of pericytes we observed, degenerating (De Jong et al. 1992) and activated, are often associated with the ability of pericytes to acquire ameboid-shaped microglial morphologies, characteristic of migrating pericytes (Dore-Duffy et al. 2000; Özen et al. 2014). In addition, numerous electron micrographs revealed the presence of the vascular segments, where the endothelium lining was detached from the BL and the spaces between were filled with electron-dense material, which was most likely the residue from degenerated projections (De Jong et al. 1992). Thinning and the disassociation of the BL from the endothelium are thought to be necessary to prepare a pocket for the extension of pericyte processes or to represent the transient residue following the retraction of a pericyte process (Baum et al. 2015). Quantitative analysis did reflect qualitative changes in the morphology of pericytes after SAH. Minocycline treatment increased the pericyte area and extended the degree of the BL surface that was covered with pericyte extensions.

The morphological deconstruction of the BL resulted in vascular leakage (Schöller et al. 2007), the consequences of which were visible in the form of swollen and degenerated astrocyte endfeet. Their enlarged sizes inevitably played an essential role in the pathological restraint of capillary sizes after SAH (Luse and Harris 1960). The degradation process of organelles and the cell membrane in swollen astrocytes began sooner than was previously reported (Prunell et al. 2005; Sehba and Friedrich 2013) and preceded astrocyte death in the present study. Traces of dead astrocytes were present in the form of significantly sizeable deteriorated areas within the perivascular zone. However, parenchymal astrocytes demonstrated increased GFAP expression, reflecting the astrogliosis process (Murakami et al. 2011; van Dijk et al. 2015), which was abated by minocycline administration. The CA results also showed the possible contributions of CD45-positive cells, but not IBA-1-positive cells, in the ultrastructural reorganization of the BL observed in SAH groups. Increased level of GFAP- (Care et al. 2022) and CD-45-positive cells could contribute to development of cerebral inflammation in chronic phase after SAH (Care et al. 2022). Results of the study on microglia/macrophage activity after 14 days from SAH induction in mice showed that minocycline inhibited neuronal cell death and inflammation. We observed decreased accumulation of CD45-positive cells in the vicinity of capillaries after 24 h from SAH induction in rats.

The presence of extracellular matrix proteins, including the MMPs, in the BL plays an inevitable role in the adjustment of the BL to dynamically changing conditions in capillaries and the brain (Rosenberg 1995; Rowe and Weiss 2008; Glentis et al. 2014). Our results did not show a significant difference in the level of the active form of MMP-2 or MMP-9 after SAH, or their mRNA expression levels (Sherchan et al. 2011; Wang et al. 2012; Vellimana et al. 2017). In addition, colocalization analysis showed the widespread colocalization of MMP-2 and MMP-9 with collagen IV. However, Pearson correlation coefficient analyses indicated very weak or no correlation between these proteins. Minocycline neuroprotective efficacy is mostly translated as its ability to inhibit enzymatically active forms of MMPs. Contrary to previous studies, minocycline administration did not significantly change the level of MMP-9 (Vellimana et al. 2017), due to widespread data points, indicating that SAH produces heterogeneous brain trauma with differing individual scopes (Thal et al. 2008; Höllig et al. 2015; Turan et al. 2017). However, when the MMP-2 was tested we observed the level of the MMP-2 active form was decreased after minocycline administration.

In the experimental tissue sections, endothelial cells were the primary source of EMMPRIN in the brain (Seulberger et al. 1990, 1992); therefore, some EMMPRIN protein immunofluorescence signal was present in the neuropil. However, the EMMPRIN signal did not originate from neurons, astrocytes, or microglia. In contrast to Hang et al. (Hang et al. 2012), we did not observe significant differences in EMMPRIN protein levels in brain tissue homogenates after SAH. In our opinion, these results suggest that, under SAH conditions, some portion of EMMPRIN becomes detached from the cellular membrane and/or was bound to opposite endothelial membranes, in a trans-homodimeric manner, requiring the missing proteins to be replaced by proteins collected in the submembrane stock (Tyler et al. 2012; Grass and Toole 2015). However, SAH also downregulated BSG2 expression; therefore, the decreased delivery of newly synthesized proteins could result in the exhaustion of the cellular stock. On the basis of the literature survey, only small noncoding mRNAs have been reported to inhibit BSG2 expression (Grass and Toole 2015).

We observed ultrastructural traits associated with transcellular route failure (Nahirney et al. 2016), including the presence of different-sized vacuoles and an abnormal number of transcellular vesicles, which can affect the gentle ion gradient balance between the blood and the brain (Reese and Karnovsky 1967). In contrast to stroke, the quantitative analysis of electron micrographs revealed a significant decrease in the number of vesicles within the endothelium after SAH. In addition, we noted the appearance of endothelial protrusions, which are considered to be an attempt by the endothelium to maintain healthy tissue integrity when cerebral oxygenation is altered (Chiang et al. 1968). Microthrombi are very important factors responsible for secondary brain injuries within the brain and are formed within the first hours after SAH ictus (Wang et al. 2021; Ye et al. 2021). We may have observed microthrombotic remnants in the form of debris in the lumen of SAH-affected capillaries in our studies, and we have considered the presence of long endothelial protrusions and debris within the lumen of capillaries to be a morphological manifestation of endothelium struggling to remove its parts by endocytosis. On the basis of the analysis, we suggest that the havoc wreaked by SAH also activated defensive mechanisms, exerted by the activated endothelium, which could be observed in the form of specific morphological changes. For example, in an attempt to limit the power of abnormal and nonselective molecule exchanges between the blood and the brain, endothelial cells decreased the number of transcellular vesicles and increased occludin and claudin-5 expression, to maintain intact TJ morphology, and increased the membrane surface by creating protrusions into the limited oxygenation environment. The endothelium morphology and vesicle numbers after minocycline injection were comparable to those for control capillaries, and debris within lumen capillaries was absent. Therefore, minocycline treatment improved the selectivity of transcellular transport and improved brain tissue oxygenation, visible as unchanged neuropil ultrastructure.

In conclusion, the current research on the low-dose administration of minocycline soon after SAH had explicitly beneficial effects on brain tissue ultrastructures, protecting capillaries and cells in the perivascular space from SAH-induced malformations. Minocycline exerts its neuroprotective function in multiple ways. On the one hand, minocycline chelate metal ions, such as Zn^2+^, which leads to MMP inhibition (Smith et al. 1999). On the other hand, minocycline consists of multiply substituted phenol ring similar to α-tocopherol (vitamin E), which is associated with minocycline’s free-radical scavenging activity in the mechanism of neuroprotection (Kraus et al. 2005). In addition, both activities are indirectly responsible for its anti-inflammatory effects. In this study, we focused on the MMP-related pathway and did not confirm a direct correlation between EMMPRIN and gelatinase or their contributions to the neuroprotective effects of low-dose minocycline administration following SAH. Nevertheless, we showed that minocycline alleviated the inflammation and reactive oxygen species (ROS)-related destructive effects on brain tissue we described. This supports the idea that the neuroprotective effects of minocycline in the SAH brain are not the result of a single mechanism but instead represent a multifactorial mosaic of effects.

## Supplementary Information

Below is the link to the electronic supplementary material.

Supplementary file1 (DOCX 24449 KB)

## Data Availability

The dataset used and/or analyzed during the current study are available from the corresponding author on reasonable request.
