# Supplementary material for: The protective effect of low-dose minocycline on brain microvascular ultrastructure in a rodent model of subarachnoid hemorrhage
Source: Histochem Cell Biol. 2022 Sep 24;159(1):91–114. doi: 10.1007/s00418-022-02150-9 (PMC9899762; doi:10.1007/s00418-022-02150-9)
Supplement: Supplementary file 1 — Supplementary file1 (DOCX 24449 KB) [file 418_2022_2150_MOESM1_ESM.docx]

**SUPPLEMENTARY MATERIAL**

**The protective effect of low-dose minocycline on brain microvascular ultrastructure in a rodent model of subarachnoid hemorrhage.**

Daria Gendosz de Carrillo ^1,2^, Sebastian Student ^3, 4^, Daniel Bula ^5^, Łukasz Mielańczyk ^2^, Małgorzata Burek ^6^, Patrick Meybohm ^6^, Halina Jędrzejowska-Szypułka ^1^

1. Department of Physiology, Faculty of Medical Sciences in Katowice, Medical University of Silesia, Katowice, Poland

2. Department of Histology and Cell Pathology, Faculty of Medical Sciences in Zabrze, Medical University of Silesia, Katowice, Poland

3. Department of Systems Biology and Engineering, Silesian University of Technology, Gliwice, Poland

4. Biotechnology Centre, Silesian University of Technology, Gliwice, Poland

5. Oncological and Reconstructive Surgery Department, Maria Sklodowska-Curie National research Institute of Oncology, Gliwice Branch, Gliwice, Poland

6. Department of Anaesthesiology, Intensive Care, Emergency and Pain Medicine, University Hospital Würzburg, Würzburg, Germany

**Short title: Minocycline protects microvasculature from SAH injury**

**Corresponding author:**

Daria Gendosz de Carrillo

Medical University of Silesia

Poniatowskiego 15

40-055 Katowice

Poland

Phone: +48501366485

Email: [dariagendosz@gmail.com](mailto:dariagendosz@gmail.com),

**Figure I**. Effects of SAH and SAH with minocycline on the colocalization between occludin or claudin-5 and collagen IV or EMMPRIN, and on the colocalization between laminin and collagen IV.

| 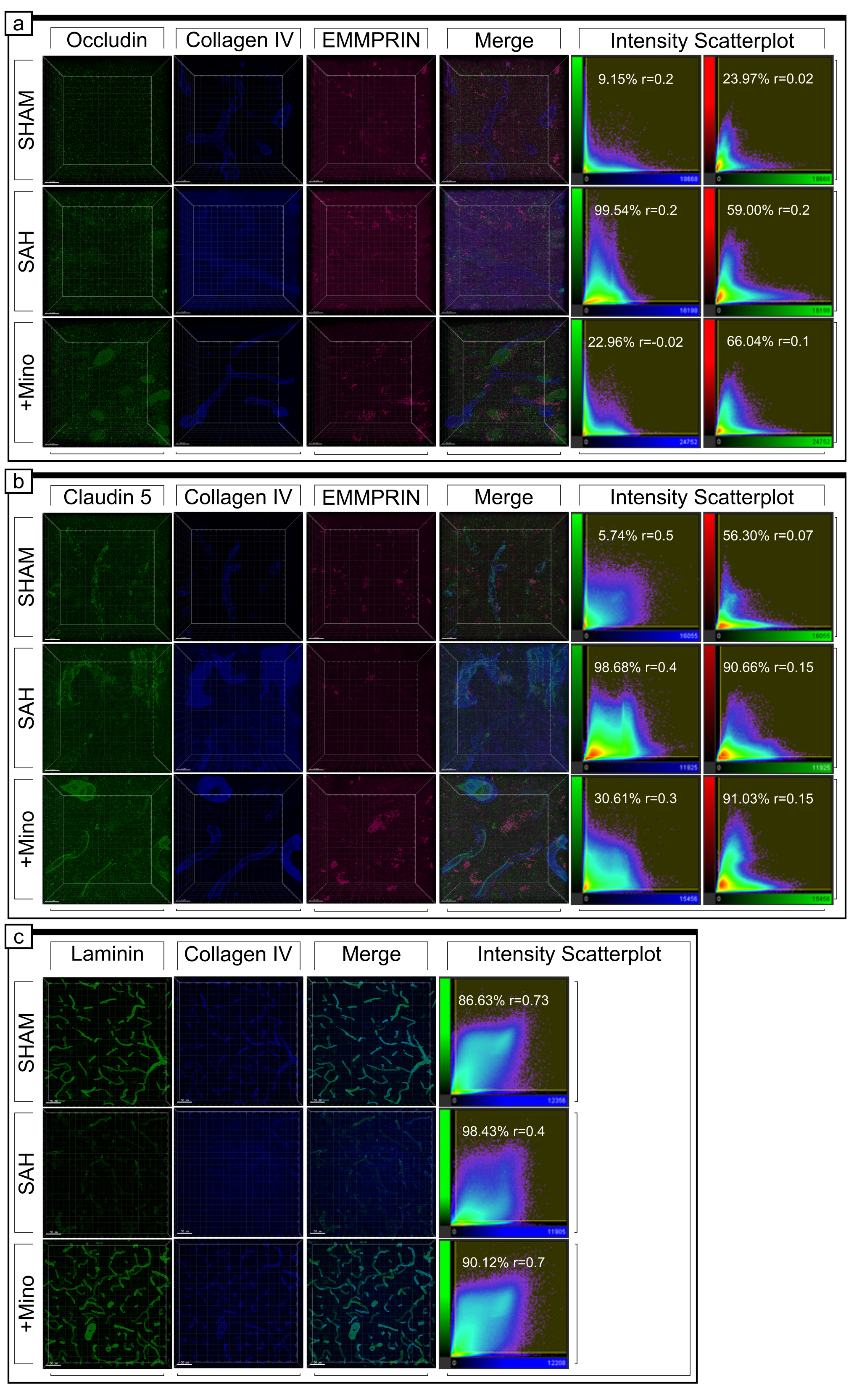 |
| --- |

**a** Confocal micrograph showing single-channel fluorescence for occludin (green, left column), collagen IV (blue, middle column), and EMMPRIN (magenta, right column) and the merged triple-channel fluorescence signal. Images are representative of the SHAM (top raw), SAH (middle raw), and SAH+Mino groups (bottom raw). Intensity scatterplots represent the fluorescence signal distributions of occludin/collagen IV (left column) and EMMPRIN/occludin (right column). **b** Confocal micrographs showing single-channel fluorescence for claudin-5 (green, left column), collagen IV (blue, middle column), and EMMPRIN (magenta, right column) and the merged triple-channel fluorescence signal. Images are representative of the SHAM (top raw), SAH (middle raw), and SAH+Mino groups (right raw). Intensity scatterplots represent the fluorescence signal distributions of claudin-5/collagen IV (left column) and EMMPRIN/claudin-5 (right column). **c** Confocal micrographs showing single-channel fluorescence for laminin (green, left column) and collagen IV (blue, middle column) and the merged double-channel fluorescence signal. Images are representative of the SHAM (top raw merge), SAH (middle raw merge), and SAH+Mino groups (right raw merge). Intensity scatterplots represent the fluorescence signal distribution of laminin/collagen IV. Values for percent of material colocalized and Pearson’s correlation coefficient (r) are included in every scatterplot. Scale bar in **a** and **b** = 10 µm; scale bar in **c** = 50 µm

**Figure II**. Effects of SAH and SAH with minocycline on colocalization between GFAP, IBA-1, and CD45 and correlation with collagen IV and on the colocalization between MMP-2 and MMP-9 and EMMPRIN or collagen IV.

| 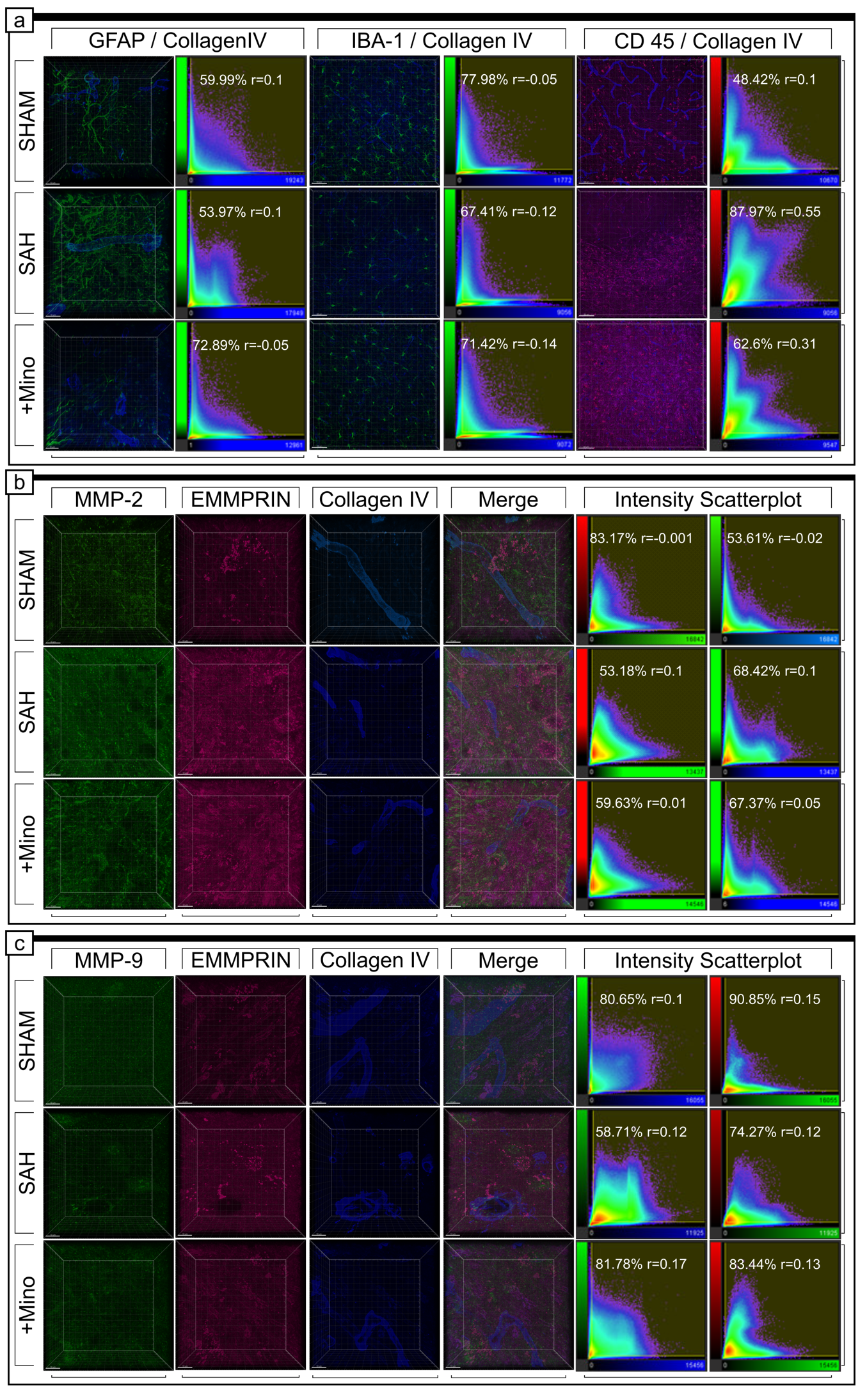 |
| --- |

**a** Confocal micrograph showing the merged double fluorescence signals for GFAP (green) and collagen IV (blue). Images are representative of the SHAM (top), SAH (middle), and SAH+Mino groups (bottom). Intensity scatterplots represent the fluorescence signal distribution of GFAP/collagen IV. **a** Confocal micrographs showing the merged double fluorescence signals for IBA-1 (green) and collagen IV (blue). Images are representative of the SHAM (top), SAH (middle), and SAH+Mino groups (bottom). Intensity scatterplots represent the fluorescence signal distribution of IBA-1/collagen IV. **a** Confocal micrographs showing the merged double fluorescence signals for CD45 (green) and collagen IV (blue). Images are representative of the SHAM (top), SAH (middle), and SAH+Mino groups (bottom). Intensity scatterplots represent the fluorescence signal distribution of CD45/collagen IV. **b** Confocal micrographs showing single-channel fluorescence for MMP-2 (green, left column), collagen IV (blue, middle column), and EMMPRIN (magenta, right column) and the merged triple channel fluorescence signal. Images are representative of the SHAM (top raw), SAH (middle raw), and SAH+Mino groups (bottom raw). Intensity scatterplots represent the fluorescence signal distribution of MMP-2/collagen IV (left column) and EMMPRIN/MMP-2 (right column). **c** Confocal micrographs showing the single-channel fluorescence for MMP-9 (green, left column), collagen IV (blue, middle column), and EMMPRIN (magenta, right column) and the merged triple channel fluorescence signal. Images are representative of the SHAM (top raw), SAH (middle raw), and SAH+Mino groups (right raw). Intensity scatterplots represent the fluorescence signal distributions of MMP-9/collagen IV (left column) and EMMPRIN/MMP-9 (right column). *Values for percent of material colocalized and Pearson’s correlation coefficient (r) are included in every scatterplot. Scale bar in **a, b, c** = 10 µm

**Figure III**. Effects of SAH and SAH with minocycline on EMMPRIN colocalization with laminin, and collagen IV, and with GFAP, IBA-1, and MAP-2.

| 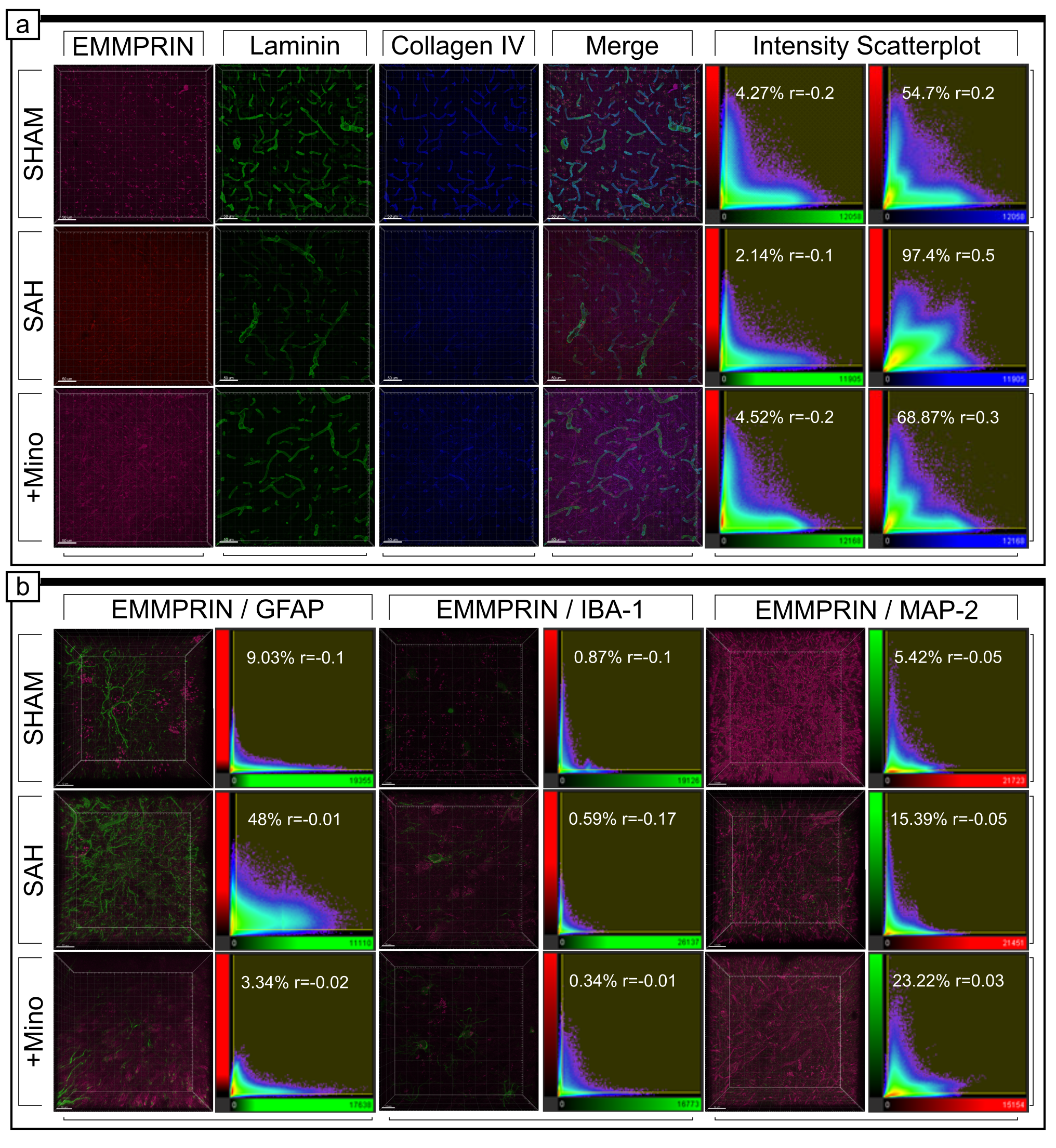 |
| --- |

**a** Confocal micrograph of single-channel fluorescence for EMMPRIN (magenta, left column), laminin (green, left column), collagen IV (blue, middle column), and the merged image of triple channel fluorescence signals. Representative images are shown for SHAM (top raw), SAH (middle raw), and SAH+Mino groups (bottom raw). Intensity scatterplots represent the fluorescence signal distributions of EMMPRIN/laminin (left column) and EMMPRIN/collagen IV (right column). **b** Confocal micrographs showing the merged double fluorescence signals for EMMPRIN (magenta) and GFAP (green). Representative images are shown for SHAM (top), SAH (middle), and SAH+Mino groups (bottom). Intensity scatterplots represent the fluorescence signal distribution of EMMPRIN/GFAP. **b** Confocal micrographs showing the merged double fluorescence signals for EMMPRIN (magenta) and IBA-1 (green). Representative images are shown for SHAM (top), SAH (middle), and SAH+Mino groups (bottom). Intensity scatterplots represent fluorescence signal distribution of EMMPRIN/IBA-1. **b** Confocal micrographs showing the merged double fluorescence signals for EMMPRIN (green) and MAP-2 (red). Representative images are shown for SHAM (top), SAH (middle), and SAH+Mino groups (bottom). Intensity scatterplots represent fluorescence signal distribution of EMMPRIN/MAP-2. *Values for percent of material colocalized and Pearson’s correlation coefficients (r) are included for every scatterplot. Scale bar in **a** = 50 µm; scale bar in **b** = 10 µm.

**Figure IV. SAH results in far-reaching neuronal damage.**


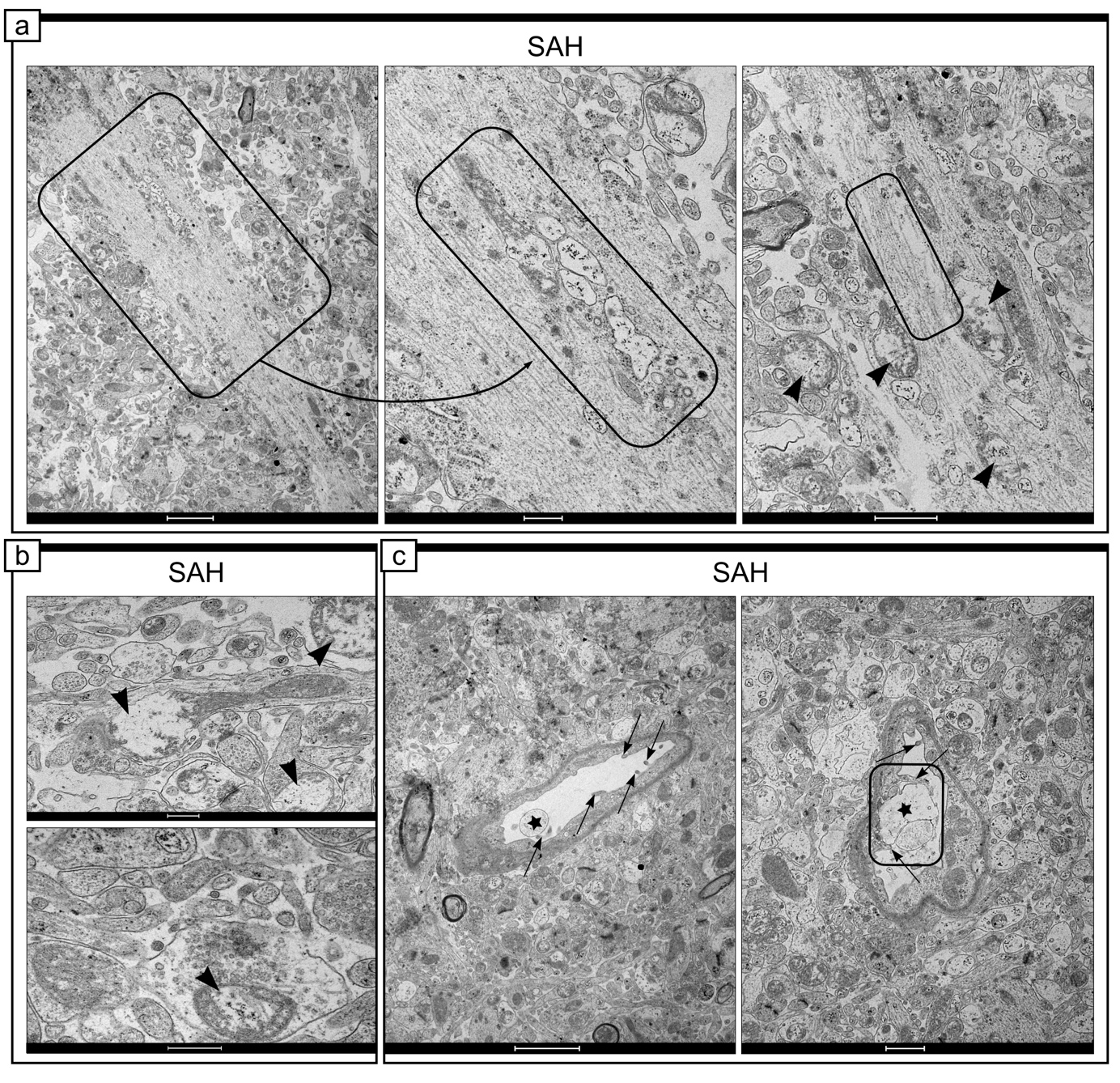


**a**, **b**, **c** Representative electron micrographs of basal cortex adjacent to SAH blood. **a** Cross-sections of neuronal fibers, containing neurofilaments with segmental loss of continuity and interrupted cell membranes, with significantly affected mitochondria (examples selected in rectangles). **b** Cristae degradation within the mitochondria (arrowhead) was also visible, in both the dendrite (b-top) and the synapse (b-bottom). **c** Long endothelial protrusions (arrow), which may initiate the engulfment of debris from the vessel lumen, such as during microvessel recanalization (examples selected in rectangles). N = 3–4 per group. Scale bar in **a left** = 2 μm, scale bar in **a middle** = 500 nm, scale bar in **a right** = 1 μm; scale bar in **b** = 500 nm; scale bar in **c left** = 2 μm, scale bar in **c right** = 2 μm.

**Figure V. SAH results in far-reaching damages to the neuropil surrounding capillaries**


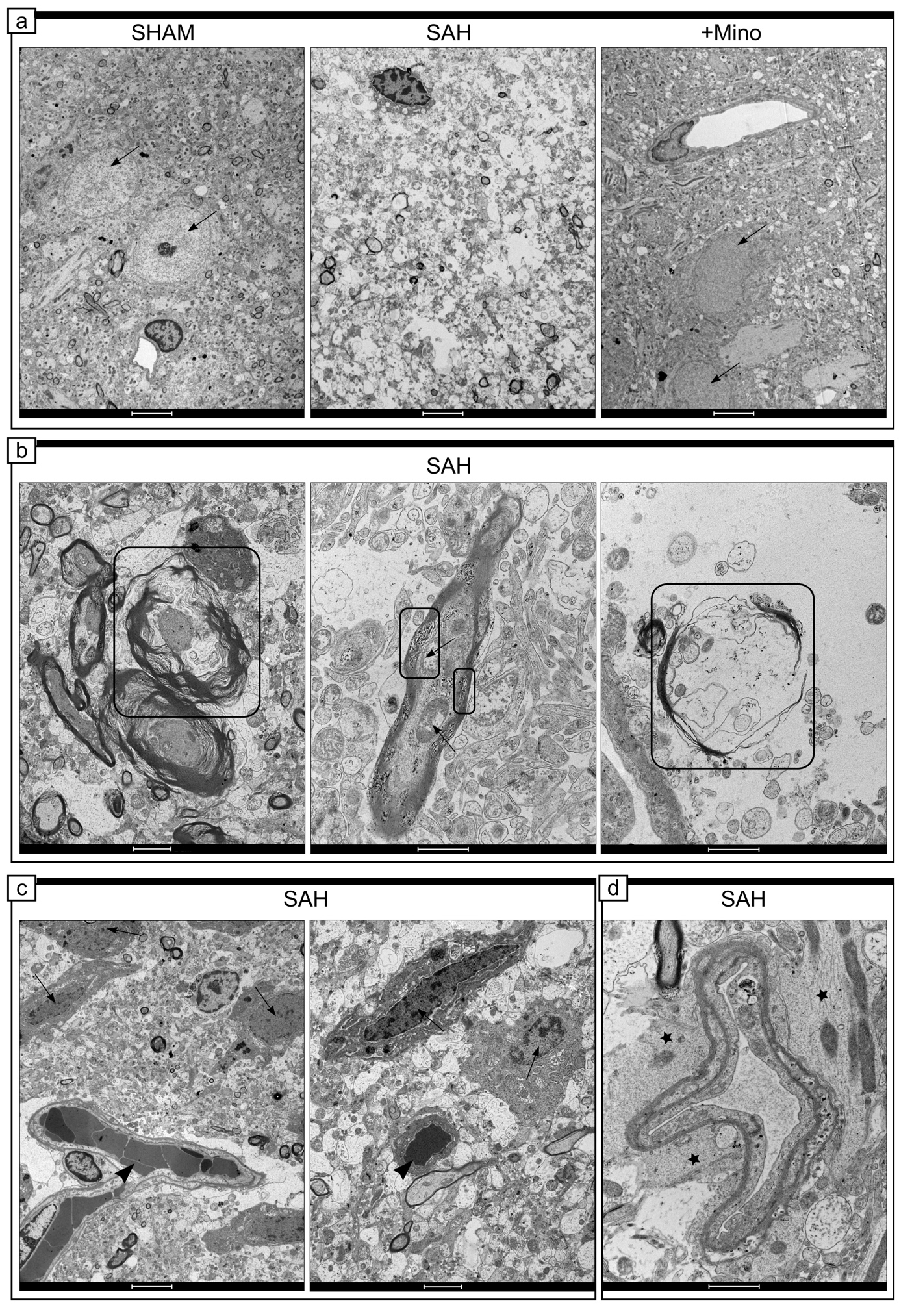


**a** Representative electron micrograph of the neuropil ultrastructure from the basal cortex, adjacent to hemorrhage blood, in SHAM, SAH and SAH+Mino samples. The neuropil of the SAH consisted of deteriorated tissue structures, with swollen astrocytes, compared with the dense arrangement of brain cells in the SHAM or SAH+Mino brain. Arrows point to perikaryon of neuronal cell. **b** Effect of SAH on axons. Selected areas enclosed by the rectangle show: flower-like myelin (left micrograph) and accelerated myelin delamination followed by its complete degradation (right micrograph). Arrows points to affected mitochondrial ultrastructures within axons. **c** Effect of SAH on axons neuronal cells. Arrows point to dark cells within close proximity to SAH-affected capillaries, which were marked here by arrowheads. **d** Micrograph represents SAH capillaries and adjacent astrocyte end-feet. Stars mark a thick net of interfilaments. N = 3–4 per group. Scale bar in **a** = 5 μm; scale bar in **b left** = 2 μm, scale bar in **b middle** and **right** = 1 μm; scale bar in **c left** = 5 μm, scale bar in **c right** = 2 μm; scale bar in **d** = 1 μm.

| **Table I.** Quantification of percent colocalized materials and Pearson’s correlation coefficients (PCC) between studied proteins in SHAM, SAH, and SAH+Mino groups related to Figure 2, 5, 6 and 8, also Figure I-III. | | | | | | | | |
| --- | --- | --- | --- | --- | --- | --- | --- | --- |
|  | SHAM | |  | SAH | |  | SAH+mino | |
|  | %MC | PCC |  | %MC | PCC |  | %MC | PCC |
| Laminin | **86.63%** | **r = 0.73** |  | **98.43%** | **r = 0.4** |  | **90.12%** | **r = 0.7** |
| Collagen IV |  |  |  |  |  |  |  |  |
|  |  |  |  |  |  |  |  |  |
| MMP-2 | 83.17% | r = -0.001 |  | 53.18% | r = 0.1 |  | 59.63% | r = 0.01 |
| Collagen IV |  |  |  |  |  |  |  |  |
|  |  |  |  |  |  |  |  |  |
| EMMPRIN | 53.61% | r = -0.02 |  | 68.42% | r = 0.1 |  | 67.37% | r = 0.05 |
| MMP-2 |  |  |  |  |  |  |  |  |
|  |  |  |  |  |  |  |  |  |
| MMP-9 | 80.65% | r = 0.1 |  | 58.71% | r = 0.12 |  | 81.78% | r = 0.17 |
| Collagen IV |  |  |  |  |  |  |  |  |
|  |  |  |  |  |  |  |  |  |
| EMMPRIN | 90.85% | r = 0.15 |  | 74.27% | r = 0.12 |  | 83.44% | r = 0.13 |
| MMP-9 |  |  |  |  |  |  |  |  |
|  |  |  |  |  |  |  |  |  |
| EMMPRIN | **54.7%** | **r = 0.2** |  | **97.4%** | **r = 0.5** |  | **68.87%** | **r = 0.3** |
| Collagen IV |  |  |  |  |  |  |  |  |
|  |  |  |  |  |  |  |  |  |
| EMMPRIN | 9.03% | r = -0.1 |  | 48% | r = -0.01 |  | 3.34% | r = -0.02 |
| GFAP |  |  |  |  |  |  |  |  |
|  |  |  |  |  |  |  |  |  |
| EMMPRIN | 5.42% | r = -0.05 |  | 15.39% | r = -0.05 |  | 23.22% | r = 0.03 |
| MAP-2 |  |  |  |  |  |  |  |  |
|  |  |  |  |  |  |  |  |  |
| EMMPRIN | 0.87% | r = -0.1 |  | 0.59% | r = -0.17 |  | 0.34% | r = -0.01 |
| IBA-1 |  |  |  |  |  |  |  |  |
|  |  |  |  |  |  |  |  |  |
| GFAP | 59.99% | r = 0.1 |  | 53.97% | r = 0.1 |  | 72.89% | r = -0.05 |
| Collagen IV |  |  |  |  |  |  |  |  |
|  |  |  |  |  |  |  |  |  |
| IBA-1 | 77.98% | r = -0.05 |  | 67.41% | r = -0.12 |  | 71.42% | r = -0.14 |
| Collagen IV |  |  |  |  |  |  |  |  |
|  |  |  |  |  |  |  |  |  |
| CD45 | **48.42%** | **r = 0.1** |  | **87.97%** | **r = 0.55** |  | **62.6%** | **r = 0.31** |
| Collagen IV |  |  |  |  |  |  |  |  |
|  |  |  |  |  |  |  |  |  |
| Occludin | **9.15%** | **r = 0.2** |  | **99.54%** | **r = 0.2** |  | **22.96%** | **r = -0.02** |
| Collagen IV |  |  |  |  |  |  |  |  |
|  |  |  |  |  |  |  |  |  |
| EMMPRIN | 23.97% | r = 0.02 |  | 59% | r = 0.2 |  | 66.04% | r = 0.1 |
| Occludin |  |  |  |  |  |  |  |  |
|  |  |  |  |  |  |  |  |  |
| Claudin 5 | **5.74%** | **r = 0.5** |  | **98.68%** | **r = 0.4** |  | **30.61%** | **r = 0.3** |
| Collagen IV |  |  |  |  |  |  |  |  |
|  |  |  |  |  |  |  |  |  |
| EMMPRIN | 56.3% | r =0.07 |  | 90.66% | r = 0.15 |  | 91.03% | r = 0.15 |
| Claudin 5 |  |  |  |  |  |  |  |  |
| Data are presented as the mean, n = 3–5 per group. %MC, percent of material colocalized, PCC, Pearson’s correlation coefficient (r). | | | | | | | | |
